# Identification and Molecular Characterization of the Homogentisate Pathway Responsible for Pyomelanin Production, the Major Melanin Constituents in *Aeromonas media* WS

**DOI:** 10.1371/journal.pone.0120923

**Published:** 2015-03-20

**Authors:** He Wang, Yunqian Qiao, Baozhong Chai, Chenxi Qiu, Xiangdong Chen

**Affiliations:** 1 State Key Laboratory of Virology, College of Life Sciences, Wuhan University, Wuhan, China; 2 Hubei Provincial Cooperative Innovation Center of Industrial Fermentation, Wuhan, China; University Paris South, FRANCE

## Abstract

The pigmentation of many *Aeromonas* species has been thought to be due to the production of a L-DOPA (L-3,4-dihydroxyphenylalanine) based melanin. However, in this study we found that although L-DOPA synthesis occurs in the high-melanin-yielding *Aeromonas media* strain WS, it plays a minor, if any, role in pigmentation. Instead, the pigmentation of *A*. *media* strain WS is due to the production of pyomelanin through HGA (homogentisate). Gene products of *phhA* (encodes phenylalanine hydroxylase), *tyrB* and *aspC* (both encode aromatic amino acid aminotransferase), and *hppD* (encodes 4-hydroxyphenylpyruvate dioxygenase) constitute a linear pathway of converting phenylalanine to HGA and disruption of any one of these genes impairs or blocks pigmentation of *A*. *media* strain WS. This HGA biosynthesis pathway is widely distributed in *Aeromonas*, but HGA is only detectable in the cultures of pigmented *Aeromonas* species. Heterologous expression of HppD from both pigmented and non-pigmented *Aeromonas* species in *E*. *coli* leads to the production of pyomelanin and thus pigmentation, suggesting that most *Aeromonas* species have the critical enzymes to produce pyomelanin through HGA. Taken together, we have identified a widely conserved biosynthesis pathway of HGA based pyomelanin in *Aeromonas* that may be responsible for pigmentation of many *Aeromonas* species.

## Introduction

Melanin is a group of negatively charged hydrophobic macromolecules formed by the enzymatic oxidation and subsequent polymerization of phenolic and/or indolic compounds [1, 2]. Production of melanin occurs in almost every taxon of living organisms ranging from bacteria to human [3, 4]. Although melanin is normally not essential for the growth of organisms, it can help the organisms compete and survive under certain environmental stress conditions, such as UV radiation and toxic free radicals [5, 6]. Melanin produced by some pathogenic microbes is also believed to protect the microbes from host defenses [7, 8]. Meanwhile, microbial melanin has multiple biotechnological applications, such as photoprotectant, antioxidant, semiconductor, energy transducer, drugs carriers, and cation exchangers [9–11].

Melanin can be classified into four categories based on the intermediates of melanogenesis: eumelanin, pheomelanin, allomelanin and pyomelanin. Eumelanin is derived from the L-DOPA, pheomelanin forms from cysteinylation of L-DOPA, allomelanin results from nitrogen-free precursors, and pyomelanin is produced from HGA [12–14]. In bacteria, melanin is usually synthesized from either L-DOPA or HGA. DOPA based melanin which contains eumelanin and pheomelanin is catalyzed by tyrosinase, which converts tyrosine to L-DOPA and then to dopaquinone. The latter then undergoes auto-oxidization and polymerization to form melanin. Production of DOPA based melanin has been described to occur in a wide range of bacteria, including *Bacillus*, *Marinomonas*, *Rhizobium*, *Streptomyces* and *Thermomicrobium* [15–19]. HGA based melanin, or the so-called pyomelanin, is carried out by the 4-hydroxyphenylpyruvate dioxygenase (HppD) which catalyzes the reaction from 4-hydroxyphenylpyruvate to HGA. The latter then auto-oxidizes to form benzoquinoneacetic acid and then self polymerizes to produce the pigment [20]. Bacteria known to synthesize HGA-based pyomelanin are *Hyphomonas*, *Pseudomonas*, *Ralstonia*, *Shewanella*, *Sinorhizobium*, *Vibrio* and *Xanthomonas* [21–25].

*Aeromonas* are rod-shaped, gram-negative, facultative anaerobic bacteria that are widely distributed in terrestrial and aquatic environments [26]. The *Aeromonas* genus currently contains 31 species (http://www.bacterio.net/-allnamesac.html), some of which are able to synthesize melanin, such as *A*. *salmonicida*, *A*. *media* and *A*. *liquefaciens* [27, 28]. However, a number of *Aeromonas* species are believed to never undergo melanogenesis, such as *A*. *allosaccharophila*, *A*. *encheleia* and *A*. *hydrophila* [29–31]. The melanin produced in *Aeromonas* had been considered to be DOPA based melanin because L-DOPA but not HGA had been detected in the bacterial cultures [27, 28, 32]. Consistent with the documents, we also detected the melanin precursor L-DOPA in the culture of a high-melanin-yielding strain *A*. *media* strain WS isolated from the East Lake, Wuhan, China. A distinct tyrosinase TyrA was also identified from this bacterium [33]. However, subsequent study found that deletion of *tyrA* from the bacterium does not significantly affect pigment production, suggesting that TyrA is not necessary for pigmentation and DOPA-based melanin is likely not the primary melanin produced by *A*. *media* strain WS [34].

In order to unravel the molecular determinants involved in the pigment formation in *A*. *media* strain WS, we screened for *A*. *media* WS mutants that were defective in pigmentation by transposon mutagenesis. Through studies of some of the isolated mutants, we found that the major melanin constituents produced by this bacterium is HGA-based pyomelanin rather than L-DOPA based melanin as previously believed. Phenylalanine 4-monooxygenase, aromatic amino acid aminotransferase and 4-hydroxyphenylpyruvate dioxygenase are important enzymes required for HGA based melanin synthesis. Through bioinformatics analysis, we found that genes encoding for these enzymes are widely distributed in *Aeromonas*, including both pigmented and non-pigmented *Aeromonas* species. However, we found that HGA is detectable in the cultures of pigmented *Aeromonas* species but not in that of the non-pigmented *Aeromonas hydrophila*, although both have functional HppD. Consistent with the result in *A*. *media* strain WS, L-DOPA is detected in the culture of *A*. *hydrophila*, indicating that the presence of L-DOPA does not correlate with pigmentation. Based on these results, we propose that pigmentation of many *Aeromonas* species, if not all of them, is due to the production of pyomelanin through HGA rather than the production of L-DOPA based melanin.

## Materials and Methods

### Bacterial strains and media

The strains used in this study are listed in Table 1. The high-melanin-yielding *A*. *media* strain WS was isolated previously from the East Lake, Wuhan, China [35]. *A*. *salmonicida*_AB98041 and *A*. *salmonicida* KACC14791 were obtained from the China Center for Type Culture Collection (CCTCC) and Korean Agricultural Culture Collection (KACC), respectively. Both of them could produce melanin when grown at 22°C but not at 30°C or above. *A*. *hydrophila*_XS91–4–1, which does not produce melanin, was a kind gift by Prof. Aihua Li of Institute of Hydrobiology, Chinese Academy of Sciences. All *Aeromonas* strains were generally cultured in Luria-Bertani (LB) medium at 30°C, but the two *A*. *salmonicida* strains were cultured at 22°C for the production of melanin. The strains of *Escherichia coli* were grown in LB at 37°C unless otherwise stated. If necessary, this medium was supplemented with kanamycin (50 μg ml^-1^), ampicillin (100 μg ml^-1^), chloramphenicol (34 μg ml^-1^) or gentamicin (50 μg ml^-1^), respectively.

**Table 1:** Bacterial strains used in this study.

Strains	Characteristic(s)	Reference or source
*A*. *media* strain WS	wild type, high-melanin-yielding strain, Amp^r^ Cm^s^	35
WS-M1/ WS-M2/ WS-M3/ WS-M4/ WS-M5/ WS-M6/ WS-M7/ WS-M8/ WS-M9/ WS-M11/	*A*. *media* strain WS with a Tn5 transposon insertion, exhibiting reduced melanin production (about 40%-70%) but with no detectable growth defect	This study
WS-M12/ WS-M14	*A*. *media* strain WS with a Tn5 transposon insertion, exhibiting reduction in both melanin production and growth capacity	This study
WS-M10/ WS-M13	*A*. *media* strain WS with a Tn5 transposon insertion, exhibiting abolished melanin production	This study
WSΔ*phhA*	*A*. *media* strain WS with a deletion of *phhA*	This study
WSΔ*tyrB*	*A*. *media* strain WS with a deletion of *tyrB*	This study
WSΔ*aspC*	*A*. *media* strain WS with a deletion of *aspC*	This study
WSΔ*tyrB*Δ*aspC*	*A*. *media* strain WSΔ*tyrB* with a deletion of *aspC*	This study
WSΔ*hppD*	*A*. *media* strain WS with a deletion of *hppD*	This study
*A*. *salmonicida*_AB98041	Pigment-producing *Aeromonas* strain	CCTCC
*A*. *salmonicida*KACC14791	Pigment-producing *Aeromonas* strain	KACC
*A*. *hydrophila*_XS91-4-1	Non-pigment-producing *Aeromonas* strain	Kindly donated by Prof. Aihua Li from Institute of Hydrobiology, Chinese Academy of Sciences
*E*. *coli* DH5α	*recA1 endA1 gyrA96 thi-1 hsdR17 supE44 relA1 ΔlacU169* (φ80*lacZ*ΔM15)	Commercially available
*E*. *coli* BL21	F^-^ *ompT hsdSB*(r_B_-m_B_^-^)*gal dcm* (DE3)	Commercially available
*E*. *coli* S17-1 (λpir)	*recA thi hsdRM* ^*+*^, λ*pir* phage lysogen RP4::Mu::Km Tn7 Tp^r^Sm^r^	38

### Conjugation and transposon mutagenesis

The plasmid pUTKm containing the Tn5 transposon, which has been reported to work in a wide range of gram-negative bacteria and exhibited no bias for any specific DNA sequence [36, 37], was chosen for random mutagenesis in *A*. *media* strain WS. Nevertheless, since the antibiotic resistance cassettes in pUTKm are not suitable for the targeting strain, its ampicillin cassette was deleted and the kanamycin cassette was replaced by a chloramphenicol cassette. The resulting transposon delivery vector was named as pTnCm, its molecular manipulation flowchart was shown in S1 Fig.

The plasmid pTnCm was first introduced into *E*.*coli* S17-1 (λpir) [38] by artificial transformation with Ca^2+^ [39]. The *E*. *coli* S17-1 (pTnCm) and *A*. *media* strain WS were cultivated in 5 ml LB-ampicillin medium and LB-chloramphenicol medium overnight, respectively. Conjugation was performed between *E*. *coli* S17-1 (pTnCm) as the donor strain and *A*. *media* strain WS as the recipient strain. The donor and recipient strain cultures were mixed in a 2 mL volume in the ratio 1:1, washed twice with 1 ml of 10% glycerol, resuspended in 50 μl of 10% glycerol, and then transferred onto 0.45-μm-pore-size membranes overlaid on LB plates. After being incubated at 30°C for approximate 12 h, the membranes were washed with 1 ml LB and the collected culture mixture was serial diluted and spread on LB agar supplemented with ampicillin and chloramphenicol to select the transposon mutants. In order to identify the possible genes involved in melanogenesis, separated transposon mutants were picked, inoculated into 96-well plates and cultured at 30°C for 72 h. Every well in the 96-well plates contained 200 μl LB-ampicillin-chloramphenicol agar medium. Then, the mutants exhibiting attenuated melanin production compared to the parent strain *A*. *media* strain WS were selected and inoculated into LB to detect the general growth (see below). The strains with no detectable growth defect relative to the level of wild-type *A*. *media* strain WS were considered as potential candidate for further study. All isolated mutants were confirmed to be *A*. *media* strain WS by sequencing the 16S rRNA and other conserved genes such as *motB* (encoding the flagellar motor protein), the gene encoding protease YbbK or the gene encoding acetyltransferase.

### Measurement of the bacterial growth ability and the melanin production

The growth capacity and the pigment production of the wild-type *A*. *media* strain WS and its mutants were all evaluated according to optical density (OD). To monitor the growth capacity of the strains, bacteria were inoculated to 25 ml of LB, incubated with shaking at 30°C at 200 rpm, and then at various periods of post-inoculation, the optical density of the cultures was determined at 600 nm (OD_600_) [40]. To quantify melanin production of the strains, the cultures from different cultivation periods were filtered through a Millipore filter (pore size, 0.45 μm) and the filter-sterilized culture supernatants were tested for their absorbance at 400 nm (OD_400_) [41].

### Identification of the Tn5 transposon insertion sites

Tn5 transposon insertion sites were identified by each of the following two methods: 1) Transposon rescue by taking advantage of the chloramphenicol acetyltransferase encoding gene within the transposon’s inverted repeats [42]. The chromosomal DNA from the strain WS Tn5 mutants was extracted and digested with *Bam*HI, *Pst*I or *Xba*I, then ligated into the vector pUC18 (Fermentas). The ligation mixtures were transformed to *E*. *coli* DH5α and spread on LB plates containing ampicillin and chloramphenicol. The obtained transformants may harbor the recombinant plasmid containing the transposon with flanking insertions from strain WS. The cloned DNA fragments were determined by automated DNA sequencing (Invitrogen). 2) Thermal asymmetric interlaced PCR (TAIL-PCR) [43] by three sequential amplification steps with primers complementary to mini-Tn5 ends and arbitrary degenerate primers (S1 Table) to clone the potential DNA flanking to the transposon. PCR products were purified by Gel Extraction Kit (OMEGA), cloned into pMD19-T vector (TAKARA) and sequenced. The interrupted sequences were analyzed by comparison of the whole genome of *A*. *media* strain WS (gi: 615550237).

### Identification of the melanin intermediates in culture supernatants by high-performance liquid chromatography (HPLC) analysis

Strains were grown in LB media at 30°C or 22°C, taken at different times, and were centrifuged (16, 000 ×g, 10 min) to remove bacterial cells. Supernatants (1 ml) were mixed with 100 μl of glacial acetic acid, clarified by centrifugation, and then stored at −20°C until assayed. The frozen samples were thawed, diluted threefold with 10 mM acetic acid, and then filtered through a Millipore filter (pore size, 0.45 μm).

Twenty microliters of filtrate was loaded directly onto an Agilent Eclipse Plus C18 reverse phase column (5 μm particle size; 4.6 by 250 mm) in an Agilent 1100 liquid chromatograph equipped with a photodiode array detector (G1315B). The mobile phase was 10 mM acetic acid-methanol (90:10 [vol/vol]). The flow rate was 1 ml min^-1^, and the eluate was monitored at 260 nm and 290 nm, as previously described [8, 32, 44]. The absorption maxima of L-DOPA and HGA are 260 nm and 290 nm, respectively. Additionally, the chromatograms of standard solutions of L-DOPA and HGA (both from Sigma) were used as references to identify the corresponding HPLC peaks.

### Identification of the melanin intermediate products by mass spectrometry (MS)

The eluting peaks of cell-free culture filtrates corresponding to standard L-DOPA and HGA peaks by HPLC were collected and further analyzed by mass spectrometry (MS). MS analysis was performed using a Thermo-Finnigan LCQ advantage ion trap mass spectrometer (San Jose, CA, USA) using an ESI interface in negative-ion mode.

### Construction of the targeted gene deletion mutants

The homologous recombination method was employed to knock out the genes which may affect pigment production, namely *phhA*, *tyrB*, *aspC*, *hppD* and *hmgA* predicted to encode phenylalanine hydroxylase, aromatic amino acid aminotransferase, aromatic amino acid aminotransferase, 4-hydroxyphenylpyruvate dioxygenase and homogentisate dioxygenase, respectively. The primers used for constructing the mutants were listed in S1 Table.

To get the WSΔ*phhA* mutant, the fragments located upstream and downstream of *phhA* gene were amplified from chromosomal DNA of strain WS with the primers phhA(S)-S/phhA(S)-A and phhA(X)-S/phhA(X)-A, respectively. The fragments obtained were 242 bp (primers phhA(S)-S/phhA(S)-A) and 255 bp (phhA(X)-S/phhA(X)-A) (S1 Table), and were then subcloned into the suicide vector pDM4 [45]. The recombinant plasmid pDM-*phhA* was transformed into *E*. *coli* S17-1 (λpir) and the transformants were selected for chloramphenicol resistance (Cm^r^). Subsequently, parental mating was used to transfer the recombinant plasmid pDM-*phhA* into *A*. *media* strain WS (Amp^r^) strains. The transconjugants with the first allelic exchange were selected on LB agar plates with ampicillin and chloramphenicol. Colonies were transferred to LB agar plates for 24 h growth, and then transferred to LB agar plates containing 15% sucrose. The *phhA* deletion mutant WSΔ*phhA* was then screened and identified by PCR.

With the same approach, individual gene (*tyrB*, *aspC*, *hppD*, *hmgA1*, *hmgA2*) deletion mutants and a double gene (*tyrB*, *aspC*) deletion mutant were also created.

### Complementation of the targeted gene deletion mutants

To complement the function of the deleted genes in the mutants (WSΔ*phhA*, WSΔ*tyrB*, WSΔ*tyrB*Δ*aspC*, WSΔ*hppD*), the DNA fragments containing the Shine–Dalgarno (SD) sequences and open reading frames (ORFs) of the target genes were amplified by PCR from chromosomal DNA of *A*. *media* strain WS. The primers were shown in S1 Table. PCR products were ligated to pBBR1MCS-5 [46]. The resulting complementary plasmids pBBR1MCS-5-*phhA*, pBBR1MCS-5-*tyrB*, pBBR1MCS-5-*aspC*, pBBR1MCS-5-*hppD* and pBBR1MCS-5-*phh*(A+B) which contained the Shine–Dalgarno (SD) sequence, complete *phhA* gene and *phhB* gene (encoding 4a-carbinolamine dehydratase, supplying cofactor for PhhA), were introduced into *E*. *coli* S17-1, then transferred to the deletion mutants by conjugation, respectively.

In order to complement *A*. *media* strain WS, the HmgA coding sequences and Shine–Dalgarno (SD) sequences were amplified from *A*. *salmonicida* _AB98041, *A*. *salmonicida* KACC14791 and *A*. *hydrophila*_XS91-4-1, then subcloned into pBBR1MCS-5, respectively [46]. The complementary plasmids pBBR1MCS-5-*hmgA*-AS, pBBR1MCS-5-*hmgA*-KACC and pBBR1MCS-5-*hmgA*-AH were transformed to *E*. *coli* S17-1, then transferred to *A*. *media* strain WS by conjugation, respectively.

### Cloning, sequence analysis of the *hppD* genes from *A*. *media* strain WS, *A*. *salmonicida*_AB98041, *A*. *salmonicida* KACC14791, *A*. *hydrophila*_XS91-4-1 and heterologous expression of HppD in *E*. *coli* BL21 (DE3)

The *hppD* gene from *A*. *media* strain WS was PCR amplified from chromosomal DNA using the primers hppD(WS)-S/hppD(WS)-A (S1 Table). The PCR product was digested with *Nde*I and *Hin*dIII and ligated to *Nde*I/*Hin*dIII-digested pET26b(+) (Novagen). Then the ligations were transformed into *E*. *coli* BL21. The transformants were confirmed by PCR. The correct colonies containing the expression plasmid pET26b(+)-*hppD*-WS were inoculated to LB with kanamycin, grown at 37°C overnight, then transferred to fresh medium. When the optical density at 600 nm reached 0.5 to 0.8, 1 mM IPTG (isopropyl—D-thiogalactopyranoside) was added to induce the expression of *hppD* [44].

Based on the GenBank sequences (gi: 142852228, gi: 507222057, gi: 117562568, gi: 569548302), the primers AS(hppD)-S / AS(hppD)-A, AS(hppD)-S / AS(hppD)-A, AH(hppD)-S /A (S1 Table) were designed to clone the *hppD* genes from *A*. *salmonicida*_AB98041, *A*. *salmonicida* KACC14791 and *A*. *hydrophila*_XS91-4-1, respectively. The resulting fragments were cloned into pMD19-T vector (TAKARA) and sequenced. It is noted that the sequences of the *hppD* genes from *A*. *salmonicida*_AB98041 and *A*. *salmonicida* KACC14791 were completely identical. The PCR products were cloned to pET26b(+) to construct expression plasmids pET26b(+)-*hppD*-AS and pET26b(+)-*hppD*-AH, transformed to *E*. *coli* BL21 and then sequenced. The following procedures were the same as above.

### RT-PCR analysis of the genes (*phhA*, *phhB*, *tyrB*, *aspC*, *hppD*) transcription in *Aeromonas* strains

The transcription of the genes (*phhA*, *phhB*, *tyrB*, *aspC*, *hppD*) involved in pyomelanin synthesis was detected with reverse transcription (RT)-PCR. Total RNA was extracted from *A*. *salmonicida*_AB98041, *A*. *salmonicida* KACC14791, *A*. *hydrophila*_XS91-4-1 and *A*. *media* strain WS, respectively, at different times using Trizol reagent (Invitrogen). The isolated RNA was reverse transcribed, utilizing PrimeScript RT reagent Kit with gDNA Eraser (TAKARA). Control experiments in which reverse transcriptase was omitted from the reaction were performed to confirm the absence of contaminating DNA in the RNA samples. The resulting cDNAs were used as PCR templates. Gene specific primers, phhA(RT)-S/phhA(RT)-A, phhB(RT)-S/phhB(RT)-A, tyrB(RT)-S/tyrB(RT)-A, aspC(RT)-S/aspC(RT)-A (S1 Table) were used to detect the transcription of *phhA*, *phhB*, *tyrB*, *aspC* in *A*. *salmonicida*_AB98041, *A*. *salmonicida* KACC14791, *A*. *hydrophila*_XS91-4-1 and *A*. *media* strain WS, respectively. Moreover, the primers hppD(AS)-S/hppD(AS)-A, hppD(AS)-S/hppD(AS)-A, hppD(AH)-S/hppD(AH)-A and hppD(WS)-S/hppD(WS)-A (S1 Table) were used to analyse *hppD* transcription in *A*. *salmonicida*_AB98041, *A*. *salmonicida*KACC14791, *A*. *hydrophila*_XS91-4-1 and *A*.*media* WS, respectively. Transcription of 16S rRNA was detected using primers 16sRNA(S)/16sRNA(A) as a positive control. All the PCR products were detected by gel electrophoresis.

## Results

### Screening and identification of the genes involved in melanogenesis in *A*. *media* strain WS by transposon mutagenesis

Bacteria usually produce melanin via either HGA or L-DOPA as intermediate [8]. Consistent with the literature [27, 28, 32], we detected L-DOPA in the culture of *A*. *media* strain WS in our previous work [35]. However, subsequent study found that deletion of *tyrA*, the gene encoding the enzyme converting tyrosine to L-DOPA, does not affect pigmentation of *A*. *media* strain WS [34], suggesting that *A*. *media* strain WS produces melanin through a non-DOPA pathway.

To identify the factors involved in pigmentation of *A*. *media* strain WS, we decided to isolate mutants that were defective in pigmentation by transposon mutagenesis. When grown in LB medium, *A*. *media* strain WS colony and culture turned black because of the production of melanin [35]. Thus, *A*. *media* strain WS mutants defective in pigmentation could be easily isolated by comparing the color of the colonies or cultures to that of the wild-type WS strain. 14 mutants showing significantly less or no pigmentation were obtained from about 20, 000 transposon insertion mutants (see materials and methods for a detailed description of the screen). As listed in Table 1, while most mutants exhibited reduced melanin production, ranging from 40% to 70% relative to the level of the wild-type strain WS, two of them, namely WS-M10 and WS-M13, lost their pigmentation ability completely (S2 Fig.). All the mutated strains grew as well as wild-type strain WS except for strains WS-M12 and WS-M14, indicating that their attenuated melanin production may be caused by the growth defects (S2 Fig.). Thus, WS-M12 and WS-M14 were not kept for further analysis.

**Fig 1:**
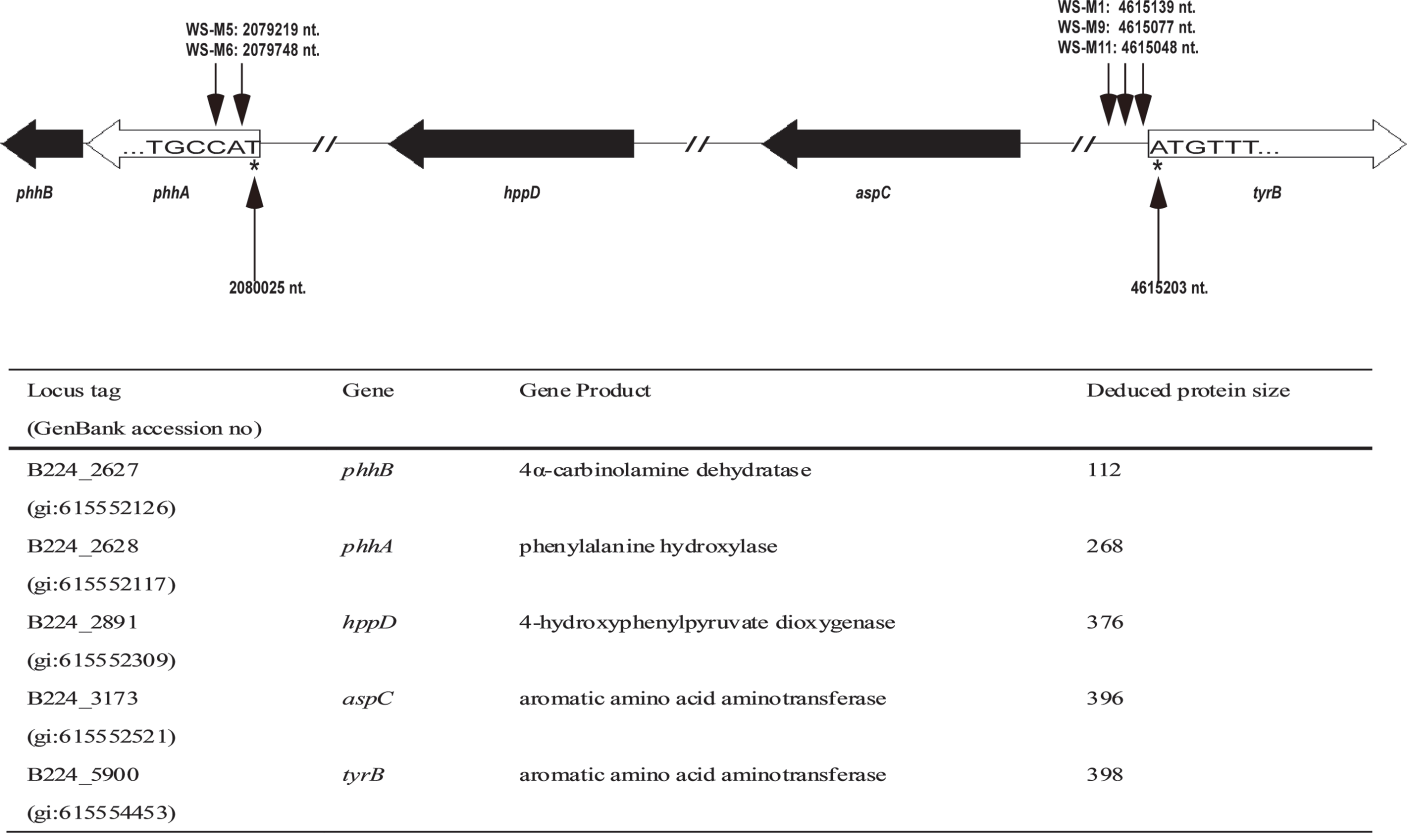
Locations of the genes in pyomealnin-synthesis pathway. The transposon insertion sites of five mutants in the genome of *A*. *media* strain WS are marked with small black arrows. The numbers after the mutant names stand for the nucleotide (nt) site just following the transposon in the genome of *A*. *media* strain WS. Locus tags, putative protein function, and the number of amino acids of the deduced proteins are listed.

As shown in Table 2, the transposon insertion sites in 9 of the 12 WS mutated strains have been identified by transposon rescue or thermal asymmetric interlaced PCR (TAIL-PCR). However, transposon-flanking sequences for 3 mutants were not obtained, including the two non-pigmented mutants, WS-M10 and WS-M13. The most frequent insertion site of the miniTn5 transposon (hit three times independently) was upstream of the start codon of *tyrB*, in mutants WS-M1, WS-M9, WS-M11 (Fig. 1). *tyrB* was predicted to encode an aromatic amino acid aminotransferase which shows 53% amino acid sequence identity to its homolog PhhC from *Pseudomonas aeruginosa* PAO1. In *Pseudomonas aeruginosa* PAO1, PhhC has been shown to transform tyrosine to 4-hydroxyphenylpyruvate, which is an intermediate in the HGA based pyomelanin biosynthesis pathway [47]. Besides *tyrB*, gene *phhA* was also hit by the miniTn5 transposon twice independently, in WS-M5 and WS-M6 (Fig. 1). *phhA* was predicted to encode a Phenylalanine 4-monooxygenase which is 50% and 62% identical to the phenylalanine hydroxylase from *Legionella pneumophila* 130b and *Pseudomonas aeruginosa* PAO1, respectively [48, 49]. Phenylalanine hydroxylase has been reported to catalyze the transformation of phenylalanine to tyrosine and promote pyomelanin production by supplying additional tyrosine [49]. The phenotypes of the transposon insertion mutants of *tyrB* and *phhA* and the functions of their homologs in other bacteria suggest that *A*. *media* strain WS may synthesize pyomelanin through the HGA pathway with PhhA providing tyrosine and TyrB providing 4-hydroxyphenylpyruvate for HGA synthesis.

**Table 2:** Melanin-deficient *A*. *media* strain WS miniTn5 transposon mutants identified in this study^a^.

Locus of insertion	No. of multiple hits	Function or product name	Phenotype	Mutants
B224_5900 (gi: 615554453)	3	Aromatic amino acid aminotransferase	Reduced	WS-M1/WS-M9/WS-M11
B224_2628 (gi: 615552127)	2	Phenylalanine 4-monooxygenase	Reduced	WS-M5/WS-M6
B224_0715 (gi: 615550742)	1	RNA methyltransferase	Reduced	WS-M3
B224_1944 (gi: 615551635)	1	type III restriction enzyme, res subunit	Reduced	WS-M7
B224_0883 (gi: 615550862)	1	Hypothetical protein	Reduced	WS-M8
B224_5895 (gi: 615554450)	1	Hypothetical protein	Reduced	WS-M2

a Three mutants (WS-M4, WS-M10, WS-M13) were unable to generate the sequences of transposon insertion sites.

In the other 4 pigmentation mutants WS-M2, WS-M3, WS-M7 and WS-M8, the miniTn5 was found to insert into gene B224_5895 encoding a hypothetical protein, gene B224_0715 encoding a RNA methyltransferase, gene B224_1944 encoding a type III restriction enzyme and B224_0883 encoding a hypothetical protein, respectively (Table 2). However, these mutants were not included in this study because of the presence of *tyrB* and *phhA* and identification of HGA in the culture supernatant of *A*. *media* strain WS (see below).

### Identification of the intermediate products involved in melanin synthesis in culture supernatants of *A*. *media* strain WS by high-performance liquid chromatography (HPLC)

Although the transposon insertion sites of the two non-pigmented mutants WS-M10 and WS-M13 could not be determined, the two mutants are great tools to determine which type of melanin is produced by *A*. *media* strain WS. As the presence of *tyrB* and *phhA* strongly suggested that *A*. *media* strain WS produces the HGA based melanin, pyomelanin, we expected to detect the presence of HGA in the culture supernatant of wild-type *A*. *media* strain WS but not in those of the two mutant strains WS-M10 and WS-M13. Methods developed to detect HGA from cultures of *Aeromonas* in previous studies have been unsuccessful [32, 35], so we followed a HPLC protocol developed by Keith KE, with slight modifications, which has successfully identified HGA in the culture of *Burkholderia cencepacia* [8]. We also analyzed cultures at different stages of pigmentation to make sure we would not miss HGA if it was only transiently available in the culture.

**Fig 2:**
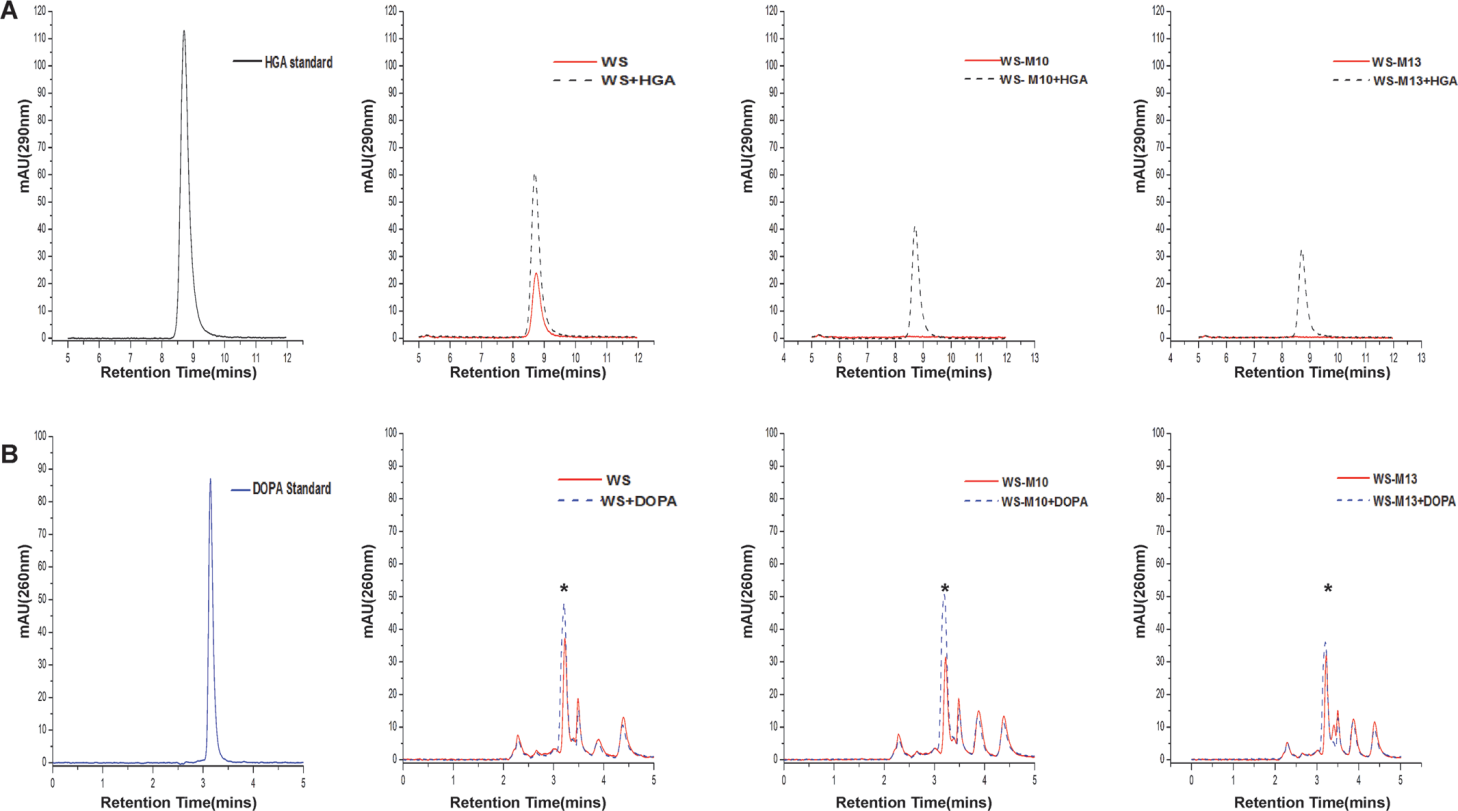
HPLC analysis. HPLC analysis of the supernatants of wild-type *A*. *media* strain WS and non-pigmented mutants WS-M10 and WS-M13. Samples were taken after 24 h of growth at 30°C in LB. (A) Analysis of HGA. (B) Analysis of L-DOPA. HGA Standard: commercial HGA (Sigma); DOPA Standard: commercial L-DOPA (Sigma). The asterisk indicates the peak of L-DOPA.

Wild-type *A*. *media* strain WS and the two mutant strains were cultured in LB liquid media with shaking at 30°C and culture samples were taken for HPLC analysis every 12 h until 72 h when melanin production reached its maximum in wild-type strain WS. Commercially available HGA was used as standard, which showed a single peak at 8.707 min (Fig. 2A). As shown in Fig. 2A and S3 Fig., a peak corresponding to HGA was identified in the 12 h, 24 h and 36 h culture supernatants of the wild-type *A*. *media* strain WS but not in those of the two mutant strains WS-M10 and WS-M13. However, it should be noted that although the peak corresponding to HGA appeared in the 12 h and 36 h culture supernatants, the values of the peak were substantially lower than that of the 24 h culture supernatant (S3 Fig.), suggesting that these two time points are either too early or too late to detect large amounts of HGA from the culture. We did not detect any HGA from the culture supernatants after the cells were grown for more than 36 h, suggesting that HGA is quickly oxidized and polymerized into pyomelanin once it is synthesized and secreted to the medium. Although these results are a little surprising given that pigment production continue to increase until 72 h, it is reasonable considering the time course of pigmentation. These results may also explain the failure to detect HGA from culture of *A*. *media* strain WS in previous studies because the time point of sampling is extremely critical for successful detection of HGA [32, 35]. Addition of HGA standard to the culture supernatant of the wild- type strain WS resulted in a peak slightly higher than the culture sample without addition of HGA standard, while addition of HGA standard to the samples of the two mutant strains WS-M10 and WS-M13 restored the peak corresponding to HGA (Fig. 2A), further confirming the correlation of pigmentation with the presence of HGA. Taken together, these results suggest that HGA is synthesized by *A*. *media* strain WS and the two non-pigmented mutants WS-M10 and WS-M13 are defective in HGA synthesis or secretion.

As L-DOPA has been detected in the culture of *A*. *media* strain WS [35], we would like to know whether L-DOPA synthesis was disrupted in the non-pigmented strains WS-M10 and WS-M13. As showed in Fig. 2B, a peak corresponding to L-DOPA was detected not only in the culture supernatant of wild-type *A*. *media* strain WS, but also in those of the two mutants (Fig. 2B and data not shown), suggesting that L-DOPA-based melanogenesis pathway exists in strain WS and the L-DOPA pathway has not been disrupted in the two non-pigmented mutants. However, this result also suggests that L-DOPA based melanogenesis plays almost no role in the pigmentation of *A*. *media* strain WS.

To further confirm that HGA and L-DOPA were produced by *A*. *media* strain WS, eluted fractions corresponding to the L-DOPA and HGA peaks from the wild-type *A*. *media* strain WS supernatant were collected for MS analysis. The spectra obtained showed the presence of molecular ion peaks at 196 and 167, similar to those in the spectrum of authentic standards, L-DOPA and HGA, respectively (S4 Fig.). Thus, although both L-DOPA and HGA are synthesized in *A*. *media* strain WS, pigmentation is largely due to HGA based pyomelanin synthesis and the L-DOPA pathway plays a very minor, if any, role in pigmentation.

### Identification of the genes involved in pyomelanin synthesis in the genome of *A*. *media* strain WS

Identification of HGA in the culture supernatant of *A*. *media* strain WS suggests that genes required for HGA synthesis must exist in the genome of *A*. *media* strain WS besides *tyrB* and *phhA*. Therefore, we searched the genome of *A*. *media* strain WS for genes whose homologs in other bacteria have been known to be involved in HGA synthesis. Analysis of the genome resulted in the identification of two other genes, *aspC* and *hppD*, which seem highly likely to be required for pyomelanin synthesis (Fig. 1). *aspC* is predicted to encode an aromatic amino acid aminotransferase that shares 50% similarity with its homolog from *Pseudomonas aeruginosa* PAO1 [47]. Aromatic amino acid aminotransferase has been reported to be involved in conversion of tyrosine to 4-hydroxyphenylpyruvate in *Pseudomonas aeruginosa* PAO1 [47]. *hppD* is assumed to encode a putative 4-hydroxyphenylpyruvate dioxygenase (HppD) whose amino acid sequence is 67%, 62%, 46% and 37% identical to HppD from *Pseudomonas aeruginosa* PAO1, *Burkholderia cenocepacia* C5424, *Shewanella colwelliana* D and *Streptomyces avermitilis* ATCC317272, respectively. The HppDs from these bacteria have been shown to catalyze the reaction from 4-hydroxyphenylpyruvate to HGA [8, 44, 50,51]. Identification of *aspC* and *hppD* confirmed our hypothesis that *A*. *media* strain WS contains all the genes required for HGA synthesis. Based on these findings, we hypothesized that PhhA converts phenylalanine to tyrosine, TyrB and AspC transform tyrosine into 4-hydroxyphenylpyruvate and HppD catalyzes the last reaction from 4-hydroxyphenylpyruvate to HGA, which in turn is further oxidized and polymerized to form pyomelanin. To confirm our hypothesis, we characterized these genes one by one in the following sections of this study.

### PhhA promotes pyomelanin production by providing additional tyrosine in *A*. *media* strain WS

**Fig 3:**
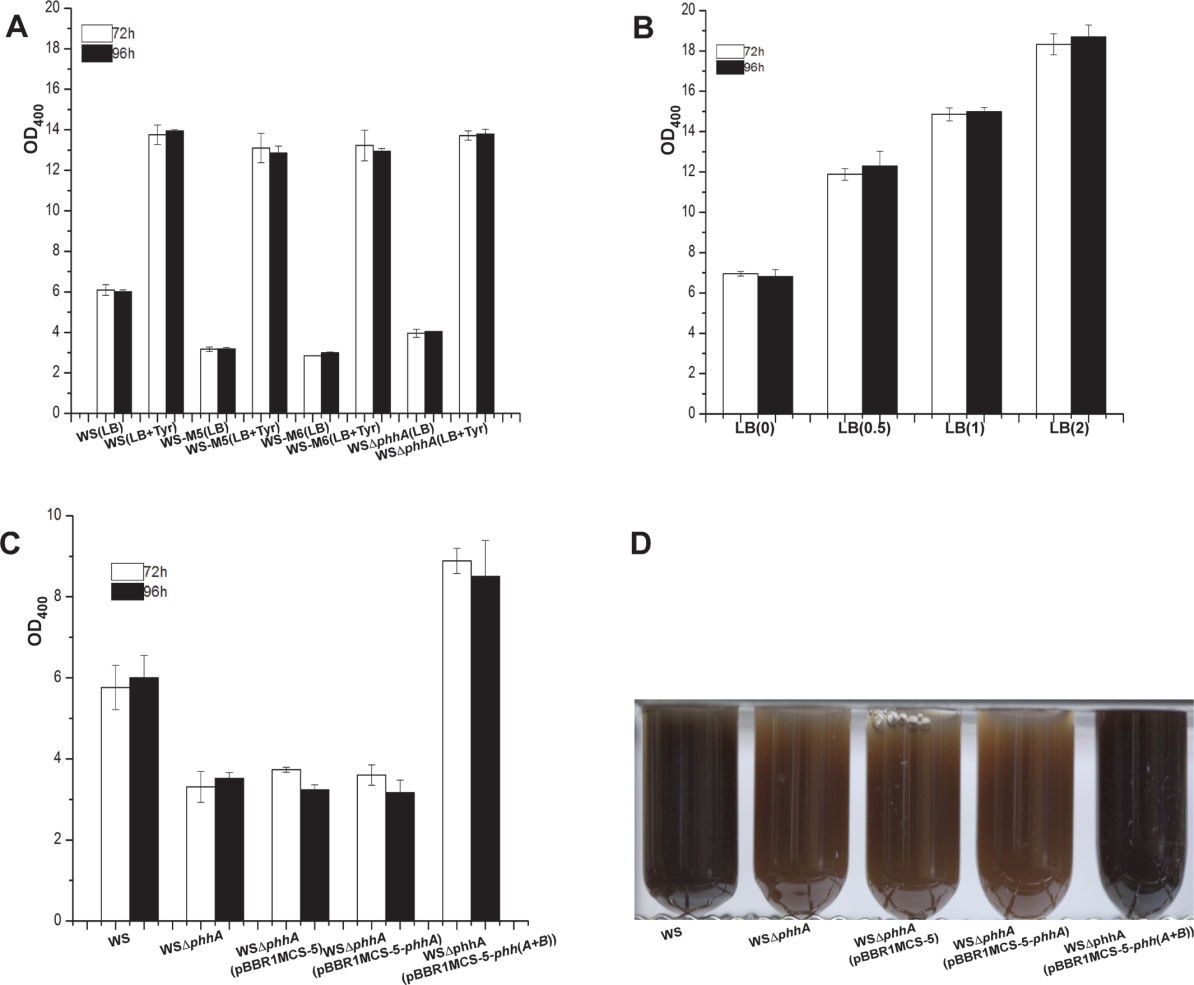
The function of *phhA* in pigmentation in *A*. *media* strain WS. (A) Wild-type *A*. *media* strain WS, *phhA* mutants WS-M5, WS-M6 and WSΔ*phhA* were inoculated into LB or LB with 1mg ml^-1^ tyrosine, and then at 72 and 96 h post-inoculation, the OD_400_ of the cultures were determined. (B) Wild-type *A*. *media* strain WS was inoculated into LB by addition of different amounts of tyrosine (0, 0.5, 1, 2 mg ml^-1^), and then at 72 and 96 h post-inoculation, the OD_400_ of the cultures were determined. (C) *A*. *media* strain WS, WSΔ*phhA* (pBBR1MCS-5), WSΔ*phhA* (pBBR1MCS-5-*phhA*) and WSΔ*phhA* (pBBR1MCS-5-*phh*(*A+B*)) were cultured in LB, and then at 72 h and 96 h post-inoculation, the OD_400_ of the cultures were determined. (D) Photographs of 72 h LB cultures of *A*. *media* strain WS, WSΔ*phhA*, WSΔ*phhA* (pBBR1MCS-5), WSΔ*phhA* (pBBR1MCS-5-*phhA*) and WSΔ*phhA* (pBBR1MCS-5-*phh*(*A+B*)). The data presented are the means and standard deviations from triplicate cultures.

In our model, PhhA promotes pyomelanin synthesis by converting phenylalanine to tyrosine. If that is true, then addition of tyrosine to the medium of the *phhA* mutant strains should restore melanin production to the wild-type level. To this end, we compared melanin production of wild-type *A*. *media* strain WS and the *phhA* mutants (WS-M5, WS-M6, WSΔ*phhA*) with or without tyrosine in the medium. As showed in Fig. 3A, all the *phhA* mutants exhibited a dramatic reduction in pigmentation when cultured without tyrosine, with strains WS-M5, WS-M6 and WSΔ*phhA* producing only 52%, 50% and 64% of that of wild-type strain WS. However, when tyrosine was present in the medium, all the *phhA* mutant strains (WS-M5, WS-M6, WSΔ*phhA*) produced as much melanin as wild-type strain WS (Fig. 3A). It is noteworthy that all the strains produced significantly more melanin in the presence of tyrosine, which is not surprising because we had showed previously that addition of tyrosine to the medium could increase melanin production by wild-type *A*. *media* strain WS and this phenomenon was confirmed again in this work [35] (Fig. 3B). These results suggest that disruption of the *phhA* gene results in a reduction of tyrosine which in turn leads to a reduction of melanin production.

To further confirm the role of *phhA*, we tried to complement the *phhA* deletion strain with a plasmid carrying *phhA*, pBBR1MCS-5-*phhA*. To our surprise, pigmentation of the *phhA* deletion mutant WSΔ*phhA* was not restored by the introduction of pBBR1MCS-5-*phhA* (Fig. 3C). As *phhA* forms an operon with the downstream gene *phhB* (predicted to encode 4a-carbinolaine dehydratase that could synthesize a cofactor tetrahydrobiopterin (BH_4_) for PhhA [52]), we speculated that the inability of pBBR1MCS-5-*phhA* to complement WSΔ*phhA* was due to a polar effect of the deletion on *phhB* (Fig. 1). Therefore, we cloned a fragment containing both *phhA* and *phhB*, and ligated it into pBBR1MCS-5. As shown in Fig. 3C and 3D, pigmentation was restored when plasmid pBBR1MCS-5-*phh*(*A*+*B*) was introduced into the *phhA* deletion strain, indicating that both *phhA* and *phhB* are important for optimal pyomelanin production in *A*. *media* strain WS.

### Both TyrB and AspC could transform tyrosine to 4-hydroxyphenylpyruvate and contribute to pyomelanin production in *A*. *media* strain WS

**Fig 4:**
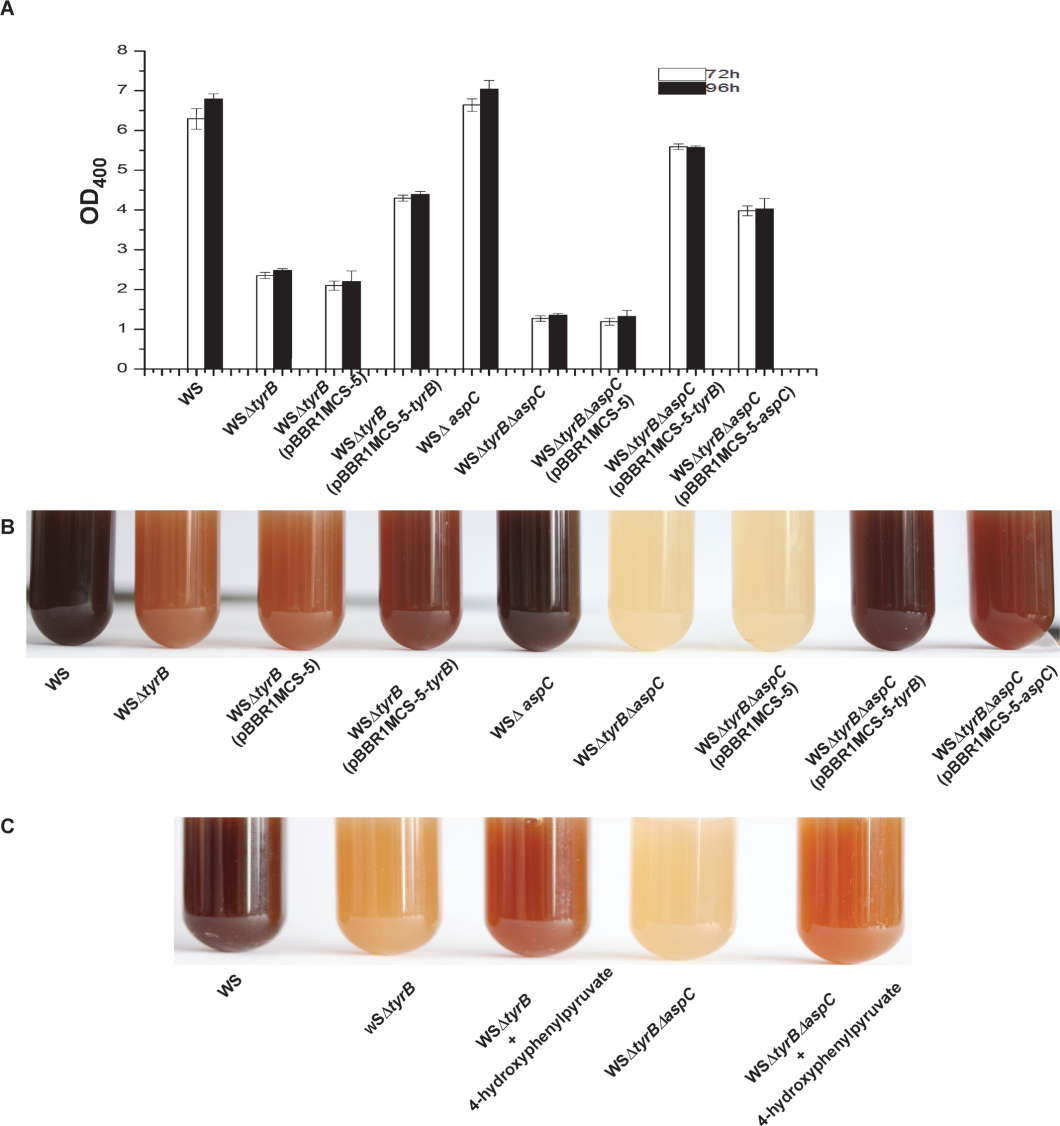
The function of *tyrB* and *aspC* in pigmentation in *A*. *media* strain WS. (A) Wild-type *A*. *media* strain WS, WSΔ*tyrB*, WSΔ*aspC*, WSΔ*tyrB*Δ*aspC*, WSΔ*tyrB* (pBBR1MCS-5), WSΔ*tyrB* (pBBR1MCS-5-*tyrB*), WSΔ*tyrB*Δ*aspC* (pBBR1MCS-5), WSΔ*tyrB*Δ*aspC* (pBBR1MCS-5-*tyrB*), WSΔ*tyrB*Δ*aspC* (pBBR1MCS-5-*aspC*) were cultured in LB, and then at 72 h and 96h post-inoculation, the OD_400_ of the cultures were determined. (B) Photographs of cultures from 72 h LB cultures of the strains. (C) Pigment production can be restored to WSΔ*tyrB* and WSΔ*tyrB*Δ*aspC* by the addition of 5 mM 4-hydroxyphenylpyruvate to the LB.

Both *tyrB* and *aspC* in strain WS are predicted to encode aromatic amino acid aminotransferase which is believed to be able to transform tyrosine into 4-hydroxyphenylpyruvate and contribute to pyomelanin production [47]. To confirm that both of these two genes are involved in pyomelanin biosynthesis in *A*. *media* strain WS, we constructed single and double deletion strains of *tyrB* and *aspC* (WSΔ*tyrB*, WSΔ*aspC*, WSΔ*tyrB*Δ*aspC*) and the effect of each deletion on pigmentation was compared. Consistent with the phenotypes of the transposon insertion mutants of *tyrB*, deletion of *tyrB* resulted in the reduction of melanin production to 37% of that of wild-type strain WS (Fig. 4A and 4B, S2 Fig.). However, deletion of *aspC* had no discernable effect on melanization (Fig. 4A and 4B). The *tyrB* and *aspC* double mutant WSΔ*tyrB*Δ*aspC* showed no sign of pigmentation after being cultured in LB for 72 h (Fig. 4B), suggesting that *aspC* is responsible for pigmentation in the absence of *tyrB*. Thus, *tyrB* and *aspC* play redundant roles in pigmentation but *tyrB* is more important than *aspC*.

The function of the two genes in pigmentation was further confirmed by the complementation experiments. As showed in Fig. 4A and 4B, while pigmentation of WSΔ*tyrB* could be restored by supplying the *tyrB* gene in *trans* on the plasmid pBBR1MCS-5, pigmentation of the double mutant WSΔ*tyrB*Δ*aspC* could only be partly recovered by a plasmid containing *tyrB* (pBBR1MCS-5-*tyrB*) or *aspC* (pBBR1MCS-5-*aspC*) (Fig. 4A and 4B). Consistent with the above result that *tyrB* is more important for pigment production, complementation of the double mutant WSΔ*tyrB*Δ*aspC* with *tyrB* always showed a better recovery of pigmentation compared to the complementation with *aspC* (Fig. 4A and 4B). Addition of 4-hydroxyphenylpyruvate to the cultures of WSΔ*tyrB* and WSΔ*tyrB*Δ*aspC* strains also partly restored pigmentation, suggesting that the pigmentation defect of the WSΔ*tyrB* mutant and the double mutant WSΔ*tyrB*Δ*aspC* is due to a reduction or the absence of 4-hydroxyphenylpyruvate (Fig. 4C). Taken together, these results confirm that TyrB and AspC converts tyrosine to 4-hydroxyphenylpyruvate for pyomelanin biosynthesis in *A*. *media* strain WS.

### HppD could convert 4-hydroxyphenylpyruvate to HGA and plays the key role in pyomelanin biosynthesis in *A*. *media* strain WS

**Fig 5:**
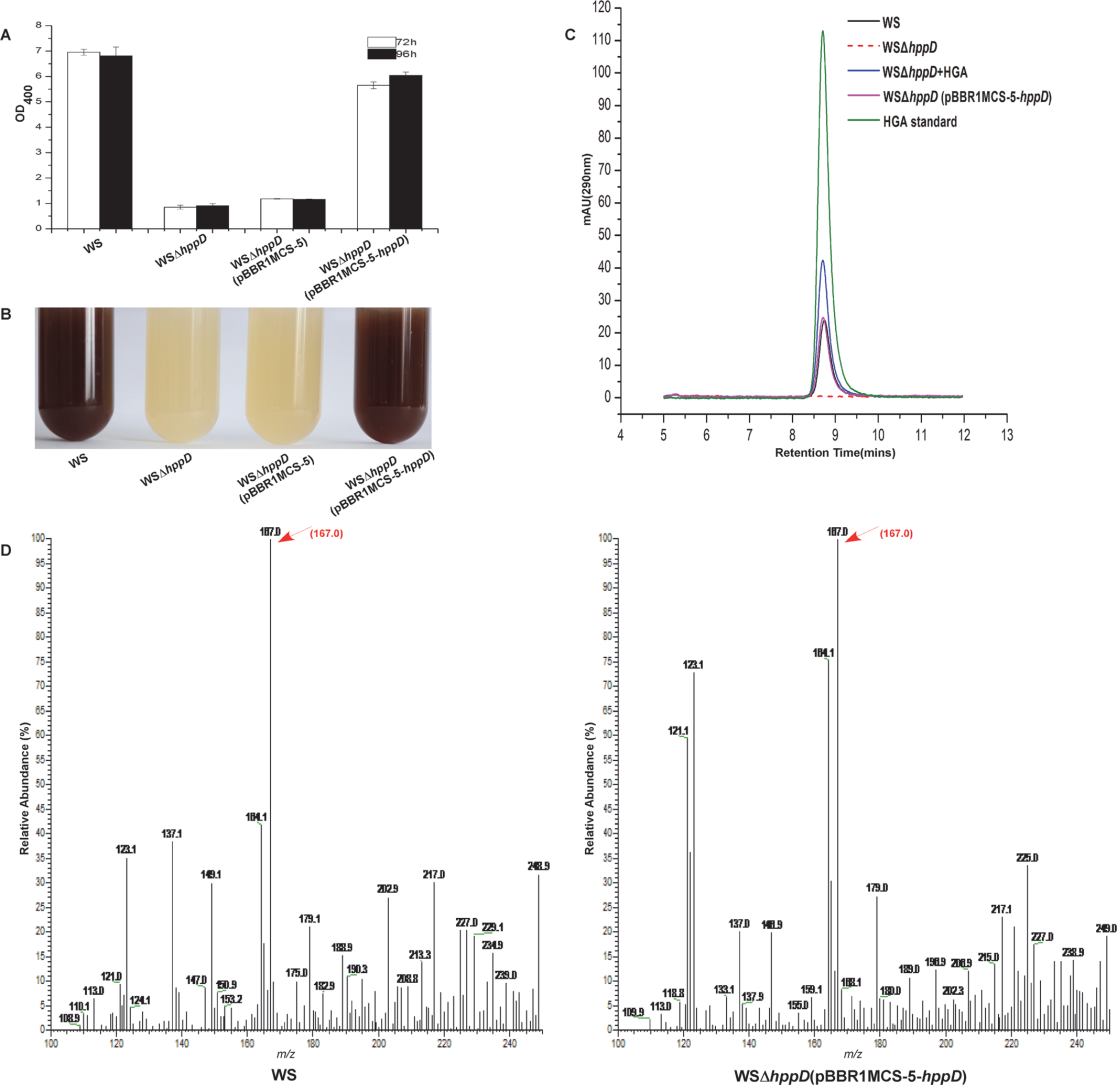
The function of *hppD* in pigmentation in *A*. *media* strain WS. (A) Wild-type *A*. *media* strain WS, WSΔ*hppD*, WSΔ*hppD* (pBBR1MCS-5) and WSΔ*hppD* (pBBR1MCS-5-*hppD*) were cultured in LB, and then at 72 h and 96 h post-inoculation, the OD_400_ of the cultures were determined. (B) Photographs of 72 h LB cultures of the strains. (C) HPLC chromatograms of culture supernatants of wild-type *A*. *media* strain WS, WSΔ*hppD* and WSΔ*hppD* (pBBR1MCS-5-*hppD*). Samples were taken after 24 h of growth at 30°C in LB. (D) MS analysis of the sample from wild-type *A*. *media* strain WS and WSΔ*hppD* (pBBR1MCS-5-*hppD*), the arrow indicates the molecular ion peaks at 167, the same to that of HGA.

*hppD* encodes a putative 4-hydroxyphenylpyruvate dioxygenase (HppD) that could transform 4-hydroxyphenylpyruvate to HGA, which then could form pyomelanin by self-oxidization and polymerization [8]. To determine the function of HppD in *A*. *media* strain WS, an *hppD* deletion strain (WSΔ*hppD*) was constructed. As shown in Fig. 5A and 5B, while the wild-type *A*. *media* strain WS produced a significant amount of pigment when grew in LB for 72 h, WSΔ*hppD* lost the ability of pigmentation completely, suggesting that *hppD* plays a crucial role in pigmentation in *A*. *media* strain WS. As expected, pigmentation of WSΔ*hppD* was restored when a plasmid carrying *hppD* was introduced into the strain (Fig. 5A and 5B).

As HppD is predicted to catalyze the reaction from 4-hydroxyphenylpyruvate to HGA, we should not be able to detect HGA in the culture supernatant when *hppD* is deleted from the chromosome. To test this, we employed HPLC to detect HGA in the 24 h culture supernatant of wild-type *A*. *media* strain WS, the deletion mutant WSΔ*hppD* and the complementary strain WSΔ*hppD* (pBBR1MCS-5-*hppD*). As shown in Fig. 5C, a peak matching the HGA standard could be detected in the culture supernatant of wild-type strain WS but not in the culture supernatant of the WSΔ*hppD*. Introduction of the plasmid pBBR1MCS-5-*hppD* into WSΔ*hppD* restored the peak corresponding to HGA in the culture supernatant (Fig. 5C). MS analysis of the elution fractions matching with the HGA peaks from wild-type *A*. *media* strain WS and WSΔ*hppD* (pBBR1MCS-5-*hppD*) showed the presence of molecular ion peaks at 167, the same to the spectrum of that of standard HGA (Sigma) (Fig. 5D). Therefore, these results strongly argue that HppD convert 4-hydroxyphenylpyruvate to HGA and plays a critical role in pigmentation in *A*. *media* strain WS.

### The HGA synthesis pathway is widely distributed in *Aeromonas*

**Fig 6:**
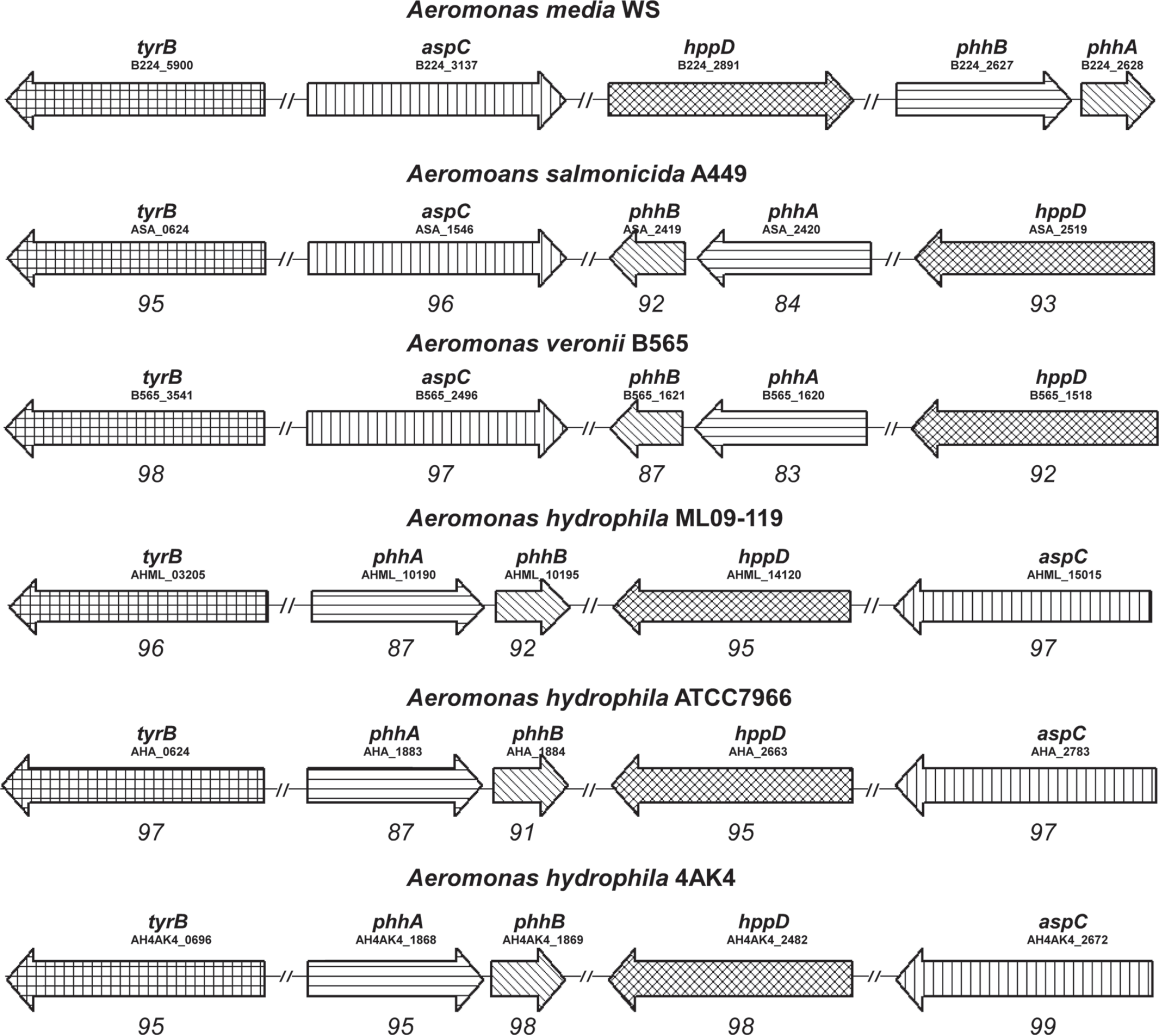
Gene organization of those genes encoding the pyomelanin synthesis pathway in *Aeromonas* strains. Genes are represented by arrows as follows: *phhA*, gene encoding the phenylalanine hydroxylase; *phhB*, gene encoding the 4a-carbinolamine dehydratase; *tyrB*, gene encoding the aromatic amino acid aminotransferase; *aspC*, gene encoding the aromatic amino acid aminotransferase; *hppD*, gene encoding the 4-hydroxyphenylpyruvate dioxygenase. The numbers beneath the arrows indicate the levels of amino acid sequence identity (expressed as percentages) between the encoded gene products and the equivalent products from *A*. *media* strain WS.

Pigmented *Aeromonas* species have been thought to synthesize melanin through the L-DOPA pathway because L-DOPA was detected in the cultures of these species [27, 28, 32]. However, our work in *A*. *media* strain WS showed that although L-DOPA is present in the culture, it plays almost no role in pigmentation. Instead, pigmentation of *A*. *media* strain WS is due to the production of pyomelanin through HGA, although HGA is only transiently detected. This suggests that the other pigmented *Aeromonas* species may also synthesize HGA based melanin rather than the predicted L-DOPA based melanin. Thus, we analyzed the genomes of many *Aeromonas* species for the presence of the genes involved in HGA synthesis in *A*. *media* strain WS. Up to present, whole genome sequencing has been finished in several *Aeromonas* species, such as *A*. *salmonicida* A449 (gi: 142849896), *A*. *veronii* B565 (gi: 328802836), *A*. *hydrophila* ML09-119 (gi: 507219248), *A*. *hydrophila* ATCC7966 (gi: 117558854) and *A*. *hydrophila* 4AK4 (gi: 569545899). As showed in Fig. 6, homologs of the genes responsible for HGA synthesis in *A*. *media* strain WS exist in all the examined *Aeromonas* species, including both pigmented and non-pigmented. This is surprising because *A*. *hydrophila* ML09-119, *A*. *hydrophila* ATCC7966 and *A*. *hydrophila* 4AK4 have been considered to be non-pigmented *Aeromonas* species. However, it is possible that *A*. *hydrophila* ML09-119, *A*. *hydrophila* ATCC7966 and *A*. *hydrophila* 4AK4 have been misclassified because we have not found the right conditions for them to produce pigment. Similar to *A*. *media* strain WS, these genes are scattered in the genomes of *Aeromonas* species with *phhA* always forming an operon with *phhB* (Fig. 6). In addition, alignments of each gene family showed that they display a high degree of identity, for example, *hppD* from *A*. *salmonicida* A449, *A*. *veronii* B565, *A*. *hydrophila* ML09-119, *A*. *hydrophila* ATCC7966 and *A*. *hydrophila* 4AK4 was 93%, 92%, 95%, 95%, 98% identical to *hppD* from *A*. *media* strain WS at the amino acid level, respectively. Based on these findings, we argued that the HGA biosynthesis pathway identified from *A*. *media* strain WS is conserved in the genus of *Aeromonas* and pigmentation of most pigmented *Aeromonas* species is likely due to the production of pyomelanin through HGA.

### Heterologous expression of *hppD* genes from different *Aermonas* species in *E*. *coli* BL21 results in pigmentation

To test our hypothesis that pigmentation of *Aeromonas* species is dependent on pyomelanin synthesis through HGA, we examined whether HppD, the critical enzymes in HGA synthesis, from several *Aeromonas* species are functional. It has been showed that heterologous expression of 4-hydroxyphenylpyruvate dioxygenase (HppD) from other bacteria in *E*. *coli* could result in pigmentation of *E*. *coli* [8, 44]. We thus cloned the *hppD* genes from the melanin producing *A*. *media* strain WS, *A*. *salmonicida*_AB98041 and *A*. *salmonicida* KACC14791, and the non-pigmented strain *A*. *hydrophila*_XS91-4-1 into pET26b(+) and expressed them in *E*. *coli* BL21. As listed in S5 Fig., the *hppD* gene from *A*. *hydrophila*_XS91-4-1 was 99%, 99%, 94% identical to *hppD* from *A*. *hydrophila* ML09-119, *A*. *hydrophila* ATCC7966, *A*. *hydrophila* 4AK4 at the amino acid level. On the other hand, the *hppD* gene seems highly conserved in the species of *A*. *salmonicida*, since the DNA sequences of the gene from the two laboratory preserved strains (*A*. *salmonicida*_AB98041, *A*. *salmonicida* KACC14791) share 100% identity with that of *A*. *salmonicida* A449.

**Fig 7:**
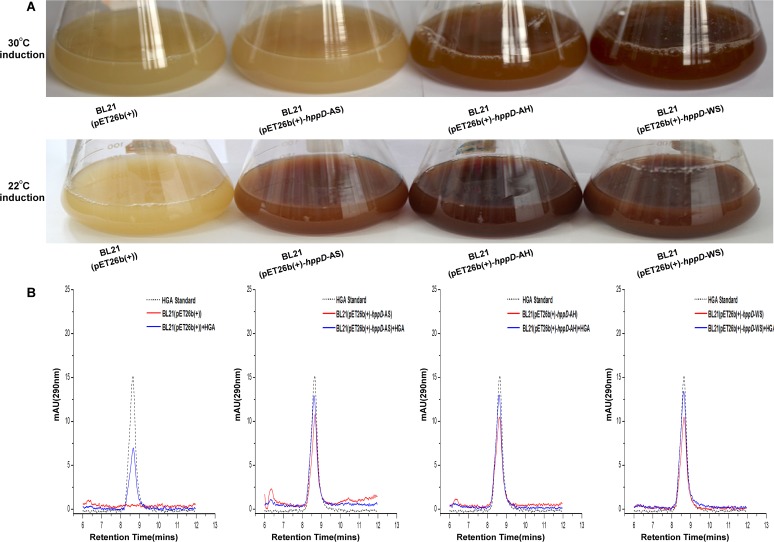
Expression of *hppD* genes from multiple *Aeromonas* sp. in *E*. *coli* BL21 results in pigmentation. (A) Photographs of *E*. *coli* BL21 (pET26b(+)), *E*. *coli* BL21 (pET26b(+)-*hppD*-AS), *E*. *coli* BL21 (pET26b(+)-*hppD*-AH) and *E*. *coli* BL21 (pET26b(+)-*hppD*-WS), incubated at 30°C and 22°C, respectively. (B) HPLC analysis of the supernatants of BL21 (pET26b(+)), BL21 (pET26b(+)-*hppD*-AS), BL21 (pET26b(+)-*hppD*-AH), BL21 (pET26b(+)-*hppD*-WS), incubated at 22°C.

As shown in Fig. 7A, when induced with 1 mM IPTG at 30°C, BL21 (pET26b(+)-*hppD*-AH) and BL21 (pET26b(+)-*hppD*-WS) produced large amounts of pigment, indicating that both the *hppD* genes from the pigmented strain WS and the non-pigmented *A*. *hydrophila* are functional. On the contrary, almost no pigment formation could be observed in the culture of BL21 (pET26b(+)-*hppD*-AS) under the same condition (Fig. 7A), although the *hppD* gene contained in the recombinant plasmid was cloned from a pigmented strain. Since the strains of *A*. *salmonicida* usually produce melanin at 20–25°C [53, 54], we changed the induction temperature accordingly. As shown in Fig. 7A, all of the three recombinant *E*. *coli* bacteria turned black when they were cultivated at 22°C, suggesting that the enzyme activity of HppD from *A*. *salmonicida* is probably temperature sensitive. In agreement with these results, we were able to detect HGA from the cultures of *E*. *coli* BL21 expressing these HppDs but not in cultures of *E*. *coli* BL21 containing just the vector (Fig. 7B). The finding that expression of *hppD* from the non-pigmented *A*. *hydrophila* in *E*. *coli* resulted in pigmentation indicates that *A*. *hydrophila* contains functional HppD, but for unknown reasons it does not pigment under the laboratory conditions.

### HGA but not L-DOPA production correlates with pigmentation in *Aeromonas*

**Fig 8:**
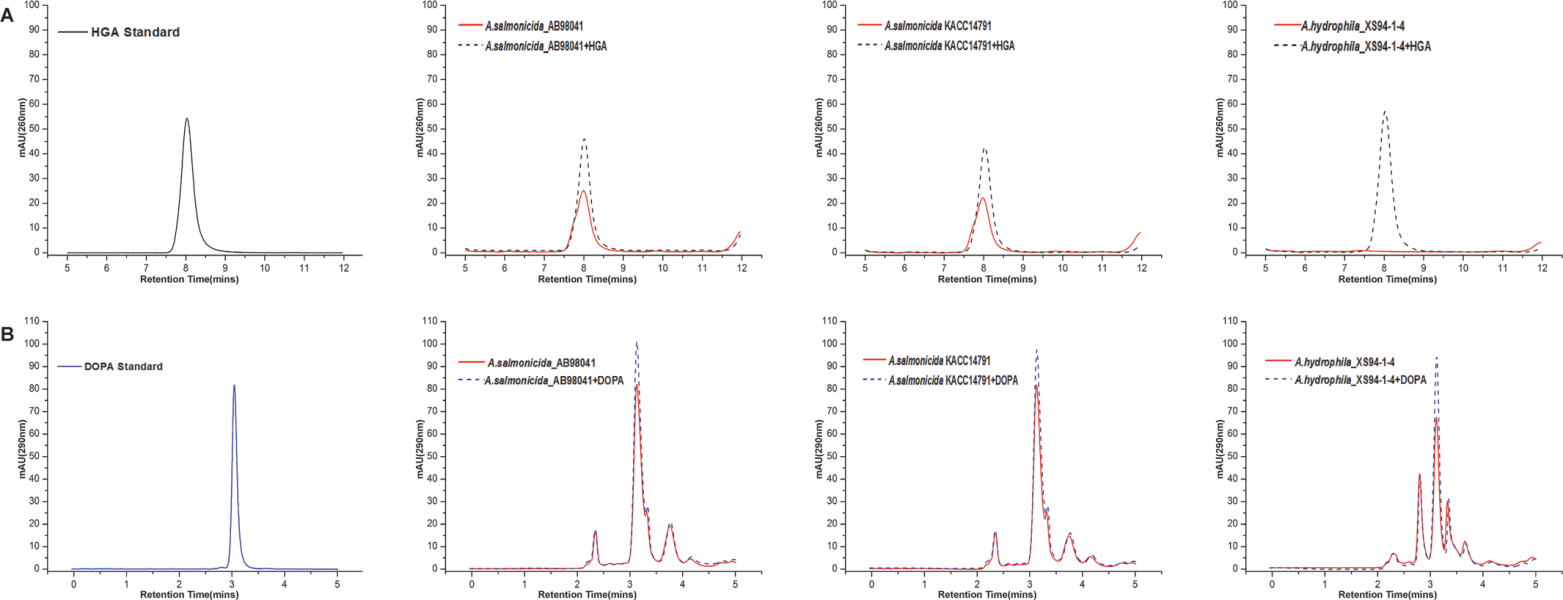
HPLC analysis. Detection of the intermediates from cultures of *A*. *salmonicida*_AB98041, *A*. *salmonicida* KACC14791 and *A*. *hydrophila*_XS91-4-1. Samples were taken after 36 h of growth in LB. (A) Analysis of HGA. (B) Analysis of L-DOPA. HGA Standard: commercial HGA (Sigma); DOPA Standard: commercial L-DOPA (Sigma).

To further test our hypothesis that pigmentation of *Aeromonas* species is dependent on pyomelanin synthesis through HGA, we examined whether HGA production correlates with pigmentation in various *Aeromonas* species. We expected to detect HGA in the cultures of pigmented *Aeromonas* species but not in those of the non-pigmented ones. To this end, we cultured the pigmented *A*. *salmonicida*, and the non-pigmented strain *A*. *hydrophila* in LB at 22°C, 30°C, respectively, and tried to detect HGA in their culture supernatants by HPLC. As showed in Fig. 8A, HGA was detected in the 36 h culture supernatants of *A*. *salmonicida*_AB98041 and *A*. *salmonicida* KACC14791 but not in that of *A*. *hydrophila* (a pattern similar to *A*. *media* strain WS was found for the two pigmented *A*. *salmonicida*, but only the data for 36 h culture supernatant was showed for simplicity). As a control, we also tested the presence of L-DOPA in the culture supernatants of these three species. Unlike HGA, L-DOPA was detected in the culture supernatants of both *A*. *salmonicida*_AB98041 and *A*. *salmonicida* KACC14791 as well as in that of *A*. *hydrophila* (Fig. 8B). Therefore, there is a strong correlation between the production of HGA and pigmentation but no correlation between the production of L-DOPA and pigmentation in many *Aeromonas* species.

### Identification of the transcription of the genes involved in pyomelanin formation in *Aeromonas* strains

The *hppD* genes cloned from pigment-producing (*A*. *salmonicida*_AB98041, *A*. *salmonicida* KACC14791, *A*. *media* strain WS) and non-pigment-producing *Aeromonas* strains (*A*. *hydrophila*_XS91-4-1) were expressed to mediate the melanin production in *E*.*coli* BL21. We want to investigate whether the differences of transcriptional level of the genes contributing to pyomelanin synthesis lead to the differences in melanin phenotype in the *Aeromonas* strains. RT-PCR analysis detected *phhA*, *phhB*, *tyrB*, *aspC*, *hppD* mRNA in *A*. *salmonicida* _AB98041, *A*. *salmonicida* KACC14791, *A*. *hydrophila*_XS94-1-4 and *A*. *media* strain WS, respectively, with expression at 12, 24, 48 and 72 h post-incubation (S6 Fig.). Taken together, these data indicated that there were no differences in the transcriptional level of *phhA*, *phhB*, *tyrB*, *aspC*, *hppD* in the pigment-producing and non-pigment-producing *Aeromonas* strains.

### The function of *hmgA* in *A*. *media* strain WS

**Fig 9:**
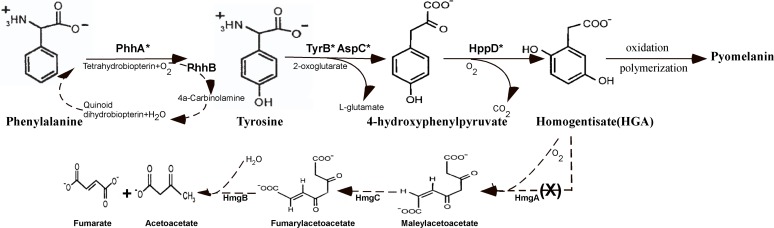
Pathway for pyomelanin synthesis and phenylalanine/tyrosine catabolism in *A*. *media* strain WS (modified from reference 49). The intermediates of the pathway are indicated. PhhA, phenylalanine hydroxylase; PhhB, 4a-carbinolamine dehydratase; AspC, aromatic amino acid aminotransferase; TyrB, aromatic amino acid aminotransferase; HppD, 4-hydroxyphenylpyruvate dioxygenase; HmgA, homogentisate dioxygenase; HmgB, fumarylacetoacetate hydrolase; HmgC, maleylacetoacetate isomerase. The asterisk indicates the location of the related genes mutated in this study.

The gene *hmgA* encodes the homogentisate dioxygenase that converts HGA to maleylacetoacetate [23] (Fig. 9). By analysis of the *hmgA* sequences from different *Aeromonas* strains, we found that the *hmgA* is interrupted into two parts, *hmgA1* and *hmgA2* in *A*. *media* strain WS (S7A Fig.). To investigate whether the genes of *hmgA1* and *hmgA2* are functional, we constructed the deletion mutants WSΔ*hmgA1* and WSΔ*hmgA2*. Pigmentation of *A*. *media* strain WS was not affected by the deletion of *hmgA1* or *hmgA2* (S7B Fig.). Moreover, we cloned the intact *hmgA* from other *Aeromonas* strains (*A*. *salmonicida* _AB98041, *A*. *salmonicida* KACC14791, *A*. *hydrophila*_XS91-4-1) and introduced them into *A*. *media* strain WS, respectively. By complementation with the *hmgA* gene, *A*. *media* strain WS exhibited reduced melanin production (S7C Fig.). Taken together, these data indicate that the *hmgA* was disrupted and lost the function in *A*. *media* strain WS.

## Discussion

In this study, we have determined that *A*. *media* strain WS produces pyomelanin through HGA rather than L-DOPA based melanin as previously thought. PhhA, TyrB and AspC, and HppD constitute a linear pathway of converting phenylalanine to HGA and this HGA biosynthesis pathway is widely conserved in the genus of *Aeromonas*, including both the pigmented and non-pigmented *Aeromonas*. Heterologous expression showed that *hppD* genes from both pigmented and non-pigmented *Aeromonas* species encode functional enzymes, suggesting that even the non-pigmented *Aeromonas* may contain necessary enzymes for pyomelanin synthesis. Moreover, we found that the presence of HGA in the culture correlates with pigmentation but there is no correlation between L-DOPA production and pigmentation. Based on these results, we propose that most of the pigmented *Aeromonas*, if not all of them, produce the HGA based pyomelanin. *Aeromonas* may also produce L-DOPA based melanin, but this kind of melanin does not seem to play a role in pigmentation. In addition, our work also suggests that there are many additional factors involved in pigmentation in *A*. *media* strain WS.

### Pigmentation of *Aeromonas* is due to the synthesis of pyomelanin through HGA

The melanogenic *Aeromonas* species had been considered to synthesize DOPA based melanin because only DOPA but not HGA was detected in the culture extracts [27, 28, 32, 35]. However, using *A*. *media* strain WS as a model we found that pigmentation of many *Aeromonas* species is likely due to the production of pyomelanin through HGA. In support of this idea, we have found that: 1) HGA is detected in the culture of wild-type *A*. *media* strain WS in the early phase of melanogenesis but not in those of the non-pigmented mutants WS-M10 and WS-M13; 2) deletion of any one of the genes involved in HGA biosynthesis impairs or blocks pigmentation of *A*. *media* strain WS dependent on the gene deleted; 3) genes encoding for enzymes required for HGA biosynthesis are widely distributed in *Aeromonas*; 4) HGA is detected in the cultures of pigmented *A*. *salmonicida* strains but not in that of the non-pigmented *A*. *hydrophila*. Similar to previous reports, we were able to detect DOPA in the cultures of *A*. *media* strain WS and cultures of pigmented *A*. *salmonicida* strains [28, 32, 35]. However, there is no correlation between the presence of L-DOPA in the culture and pigmentation of *Aeromonas* because L-DOPA was identified at high levels in the cultures of the non-pigmented *A*. *media* strain WS mutants and the non-pigmented *A*. *hydrophila*. Therefore, these results suggest that pigmentation of many *Aeromonas* species is largely due to the production of pyomelanin through HGA rather than the production of L-DOPA based melanin.

One of the reasons that many pigmented *Aeromonas* species had been considered to synthesize melanin through L-DOPA is because HGA had never been detected in the cultures of *Aeromonas* species before this study [27, 28, 32]. We also failed to detect HGA from the culture of *A*. *media* strain WS in a previous study [35]. However, the identification of *phhA* and *tyrB* from the screen for mutants defective in pigmentation led us to believe that *A*. *media* strain WS produces HGA based melanin because homologs of *phhA* and *tyrB* have been found to be involved in HGA synthesis in Pseudomonas aeruginosa PAO1 and *Legionella pneumophila* 130b [47, 49]. Following a HPLC protocol developed by Keith KE to detect HGA in the culture of *Burkholderia cencepacia* [8], we successfully detected HGA in the culture of wild-type *A*. *media* strain WS but not in those of the non-pigmented mutants. Analysis of cultures at different stages of melanogenesis appears to be critical for our success in detecting HGA because HGA is only detectable in the culture of the wild-type strain WS in the early phase of pigmentation (12 to 36 h). It should be noted that although HGA is present in the culture of *A*. *media* strain WS when it is grown in LB for 12 h or 36 h, the level of HGA is very low compared to that at 24 h, a time point pigment starts to appear in the culture. This result may explain why HGA has never been identified in the cultures of *Aeromonas* species because only samples from late exponential-phase were taken for analysis in the previous studies [32, 35]. We also successfully identified HGA from the cultures of pigmented *A*. *salmonicida* but not in that of non-pigmented *A*. *hydrophila*. Thus, we believe that previous failures to identify HGA from the cultures of pigmented *Aeromonas* are due to the low presence of HGA in the cultures and the way the experiments were performed.

### A widely conserved pyomelanin synthesis pathway in *Aeromonas*

With the characterization of *phhA*, *tyrB*, *aspC* and *hppD*, a pathway of HGA based pyomelanin production in *A*. *media* strain WS is established: 1) phenylalanine is transformed into tyrosine by phenylalanine 4-monooxygenase (PhhA) and 4a-carbinolamine dehydratase (PhhB), the latter provides the cofactor for the reaction; 2) tyrosine is then converted to 4-hydroxyphenylpyruvate by two aromatic amino acid aminotransferases (TyrB and AspC); 3) 4-hydroxyphenylpyruvate is transformed into HGA by 4-hydroxyphenylpyruvate dioxygenase (HppD); 4) the secreted HGA spontaneously polymerizes to pyomelanin (Fig. 9). In this pathway, PhhA is helpful but not necessary for melanin production because deletion of it does not eliminate pigmentation of *A*. *media* strain WS. TyrB and AspC play a redundant role in converting tyrosine into 4-hydroxyphenylpyruvateas as deletion of *tyrB* impairs pigmentation but deletion of both *tyrB* and *aspC* results in a complete loss of pigmentation. It is generally believed that there is only one aromatic amino acid aminotransferase in the pyomelanin synthesis pathway [8, 47, 49], but our work suggests that there could be more than one enzyme catalyzing the transformation of tyrosine to 4-hydroxyphenylpyruvate in pyomelanin producing bacteria. HppD appears to be the most critical enzyme in this pathway because its deletion results in the complete abrogation of pigmentation. This is not surprising as 4-hydroxyphenylpyruvate dioxygenase has been found to be essential for melanin production in *Shewanella colwelliana* and *Pseudomonas* sp. And it is ubiquitously present among pyomelanogenic microorganisms [51, 55, 56].

Genomic analysis indicates that the identified HGA synthesis pathway is widely present in *Aeromonas*, including both the pigmented and non-pigmented *Aeromonas* species. In agreement with this, heterologous expression of HppD from multiple *Aeromonas* species in *E*. *coli* results in pigmentation of *E*. *coli*, suggesting that HppDs from these bacteria are functional. Moreover, HGA is also detected in the cultures of several pigmented *Aeromonas* species. Thus, we propose that this HGA based pyomelanin synthesis pathway is widely conserved in *Aeromonas*.

### Regulation of pigmentation in *Aeromonas*

In addition to mutants of *phhA* and *tyrB*, we have also isolated additional *A*. *media* strain WS mutants that are impaired or defective in pigmentation. Although we were unable to determine the transposon insertion sites of the two non-pigmented mutants, WS-M10 and WS-M13, our pilot analysis suggests that the pigmentation defect of these two mutants may be due to the absence of HppD because addition of a plasmid carrying *hppD* into these two mutants restored pigmentation (data not shown). PCR and sequencing analysis of the genomic region around *hppD* showed that the *hppD* gene and its regulatory sequences are intact (data not shown). However, the transcript of *hppD* is not detectable in these two mutants (data not shown), suggesting that a critical transcription factor required for *hppD* expression is likely disrupted by the transposon in these two mutants. Efforts to identify the transposon insertion sites of these two mutants are ongoing, and we believe that we will learn more about the transcription regulation of pigmentation in *A*. *media* strain WS once we identify the insertion sites.

Besides these two non-pigmented mutants, the other pigmentation mutants have the miniTn5 transposon inserted in genes encoding hypothetical proteins or proteins that have never been suggested to be involved in HGA synthesis, indicating that there are many factors contributing to the regulation of pigmentation in *A*. *media* strain WS. Considering the potential functions of melanin for microbes, one should not be surprised to see that many other factors might affect melanin production in addition to the enzymes involved in biosynthesis. A recent study in *Pseudomonas aeruginosa* identified 26 genes contributing to pyomelanogenesis, including genes encoding ABC transporter, transcriptional regulator and hypothetical proteins [57]. Some of the genes identified in *P*. *aeruginosa* overlaps with those identified in this study, such as *phhA*, *tyrB* and *hppD*, which are conserved enzymes required for HGA biosynthesis. However, a number of genes seem to be unique to each screen, possibly reflecting the different lifestyles of the two bacteria. It will be interesting to determine how the genes identified in our study affect melanin production in *A*. *media* strain WS in the future.

One surprising finding from our study is that even the non-pigmented *A*. *hydrophila* contains the HGA synthesis pathway. Heterologous expression of HppD from this bacterium in *E*. *coli* leads to pigmentation of *E*. *coli*, suggesting that the genes in the HGA synthesis pathway may encode functional enzymes. However, we were unable to detect HGA in the culture of *A*. *hydrophila*, suggesting that HGA is not synthesized or not secreted in this bacterium. One possible reason why *A*. *hydrophila* does not produce pigment could be that the genes in the HGA synthesis pathway are not transcribed. However, RT-PCR showed otherwise (S6 Fig.). Another possibility is that HGA is quickly broken down by HGA oxidase, HmgA, because pyomelanin production not only depends on the synthesis of HGA, but also relies on a reduced ability of the bacterium to degrade HGA. Inactivation of *hmgA* has been reported to be responsible for the hyperproduction of pyomelanin in many bacteria and eukaryotes [58, 59]. In fact, we found that the hyperproduction of melanin by *A*. *media* strain WS was partly due to the inactivation of *hmgA*. While the *hmgA* genes in most of the *Aermonas* species are intact, the *hmgA* gene in *A*. *media* strain WS is disrupted into two parts by a gene in opposite orientation (S7A Fig.). Deletion of the two disrupted *hmgA* parts, named *hmgA1* and *hmgA2*, did not affect pigmentation, but introduction of the *hmgA* gene from *A*. *salmonicida* or *A*. *hydrophila* into *A*. *media* strain WS resulted in a 20% reduction of pigmentation (S7B and S7C Fig.). As *A*. *hydrophila* has functional *hmgA*, it is possible that HGA is produced but that it is quickly degraded by HmgA such that it does not pigment. However, this doesn’t seem to be the case, given that *A*. *salmonicida* still pigments even though it encodes functional HmgA. Moreover, HmgA activity is controlled by many factors, such as iron concentration [60, 61].

One interesting phenomenon we observed when the *hppD* gene from *A*. *salmonicida* is expressed in *E*. *coli* BL21 is that HppD only results in pigmentation of *E*. *coli* at 22°C but not at 30°C. Previous study has reported that the optimum growth temperature for *A*. *salmonicida* to produce melanin was 20–25°C and the bacterium lost the ability to produce pigment when it was cultured at supra-optimal temperature, i.e., 30–37°C [54]. Thus, it is possible that the enzymes required for HGA synthesis in *A*. *hydrophila* require a certain condition, for example, a specific temperature or pH, to be active. It is also possible that *A*. *hydrophila* is able to synthesize HGA, but HGA is not secreted into the medium due to a lack of appropriate transporter such that no pigmentation is observed. As *A*. *hydrophila* infects many organisms, including animals and humans, it may only synthesize melanin when it encounters the hosts given that melanin production has also been associated with virulence and pathogenicity in many pathogenic microbes [3, 4].

It is noteworthy to mention that the biological functions of melanin in many pigmented *Aeromonas* have never been studied, although pigmentation of *Aeromonas* has been known for a long time [31]. Melanin produced by many microorganisms has been suggested to protect the microbes from UV radiation, reactive oxygen species and host immune defense. Using the non-pigmented mutants of *A*. *media* strain WS constructed in this study, we should be able to determine whether pyomelanin has a role for *Aeromonas* under such stressed conditions. Lastly, previous studies from our lab have found that the melanin produced by *A*. *media* strain WS has many potential applications, such as photoprotection for pesticide [35], therefore, identification of the pyomelanin biosynthesis pathway provides a starting point for molecular manipulations of *A*. *media strain* WS to increase melanin production for industrial use.

## Supporting Information

S1 Fig Construction of pTnCm. Arrows and boxed text indicate the construction manipulations. The chloramphenicol resistance cassette from pBeloBAC11 is represented by an open arrow. All plasmids are drawn to scale. IR, inverted repeat.
(DOC)

S2 Fig 14 mutants with attenuated melanin production. (A) Photographs of cultures from 72 h LB cultures of wild-type *A*. *media* strain WS and the 14 transposon mutants (WS-M1, WS-M2, WS-M3, WS-M4, WS-M5, WS-M6, WS-M7, WS-M8, WS-M9, WS-M10, WS-M11, WS-M12, WS-M13, WS-M14). (B) Wild-type *A*. *media* strain WS and the 14 transposon mutants were cultured in LB, and then at 72 h post-inoculation, the OD_400_ of the cultures were determined. (C) Wild-type *A*. *media* strain WS and the 14 transposon mutants were cultured in LB, and then at 72 h post-inoculation, the OD_600_ of the cultures were determined.
(DOC)

S3 Fig HPLC analysis. Detection of HGA from cultures of wild-type *A*. *media* strain WS and non-pigmented mutants WS-M10 and WS-M13. Samples were taken at 12-h intervals until 72 h at 30°C in LB. (A) Analysis of the culture from wild-type *A*. *media* strain WS. (B) Analysis of the culture from non-pigmented mutant WS-M10. (C) Analysis of the culture from non-pigmented mutant WS-M13. The asterisk indicates the peak of HGA.
(DOC)

S4 Fig MS analysis. (A) MS analysis of an authentic L-DOPA sample. (B) MS analysis of the sample from culture of wild-type *A*. *media* strain WS. (C) MS analysis of an authentic HGA sample. (D) MS analysis of the sample from culture of wild-type *A*. *media* strain WS.
(DOC)

S5 Fig HppD primary structure. The primary structure and conservation of HppD from *A*. *hydrophila*_XS91-4-1 compared to that from *A*. *hydrophila* ML09-119, *A*. *hydrophila* ATCC7966, *A*. *hydrophila* 4AK4. Amino acids depicted in gray are little conserved.
(DOC)

S6 Fig Transcription of *phhA*, *phhB*, *tyrB*, *aspC* and *hppD*. Expression of *phhA*, *phhB*, *tyrB*, *aspC* and *hppD* transcripts in *A*. *salmonicida*_AB98041, *A*. *salmonicida* KACC14791, *A*. *hydrophila*_XS94-1-4 and *A*. *media* strain WS, which were cultivated in LB at 30°C for 12, 24, 48 and 72 h cultivation. And then RT-PCR was done using primers that amplify the specific transcripts. That the PCR products obtained resulted from mRNA templates was confirmed by the lack of product obtained when the PCR did not incorporate reverse transcriptase (- RT). PCR products obtained from genomic DNA appear in the left-most lane, indicating that the mRNAs observed are full-length. RT-PCR analysis of 16S rRNA served as a positive control.
(DOC)

S7 Fig The function of *hmgA* in pigmentation in *A*. *media* strain WS. (A) The *hmgA* gene in *A*. *media* strain WS is interrupted into two parts, *hmgA1* and *hmgA2*. *hmgA*, gene encoding the homogentisate dioxygenase. B224_2888, gene encoding a transposase. (B) Wild-type *A*. *media* strain WS, WSΔ*hmgA1*, WSΔ*hmgA2* were cultured in LB, and then at 72 h and 96 h post-inoculation, the OD_400_ of the cultures were determined. (C) Wild-type *A*. *media* strain WS, WS (pBBR1MCS-5), WS (pBBR1MCS-5-*hmgA-*AS), WS (pBBR1MCS-5-*hmgA-*KACC) and WS (pBBR1MCS-5-*hmgA-*AH) were cultured in LB, and then at 72 h and 96 h post-inoculation, the OD_400_ of the cultures were determined.
(DOC)

S1 Table Primers used in this study. (DOC)
