# Supplementary material for: Identification and Molecular Characterization of the Homogentisate Pathway Responsible for Pyomelanin Production, the Major Melanin Constituents in Aeromonas media WS
Source: PLoS One. 2015 Mar 20;10(3):e0120923. doi: 10.1371/journal.pone.0120923 (PMC4368426; doi:10.1371/journal.pone.0120923)
Supplement: S1 Fig — Arrows and boxed text indicate the construction manipulations. The chloramphenicol resistance cassette from pBeloBAC11 is represented by an open arrow. All plasmids are drawn to scale. IR, inverted repeat. (DOC) [file pone.0120923.s001.doc]

**Figure S1. Construction of pTnCm.**


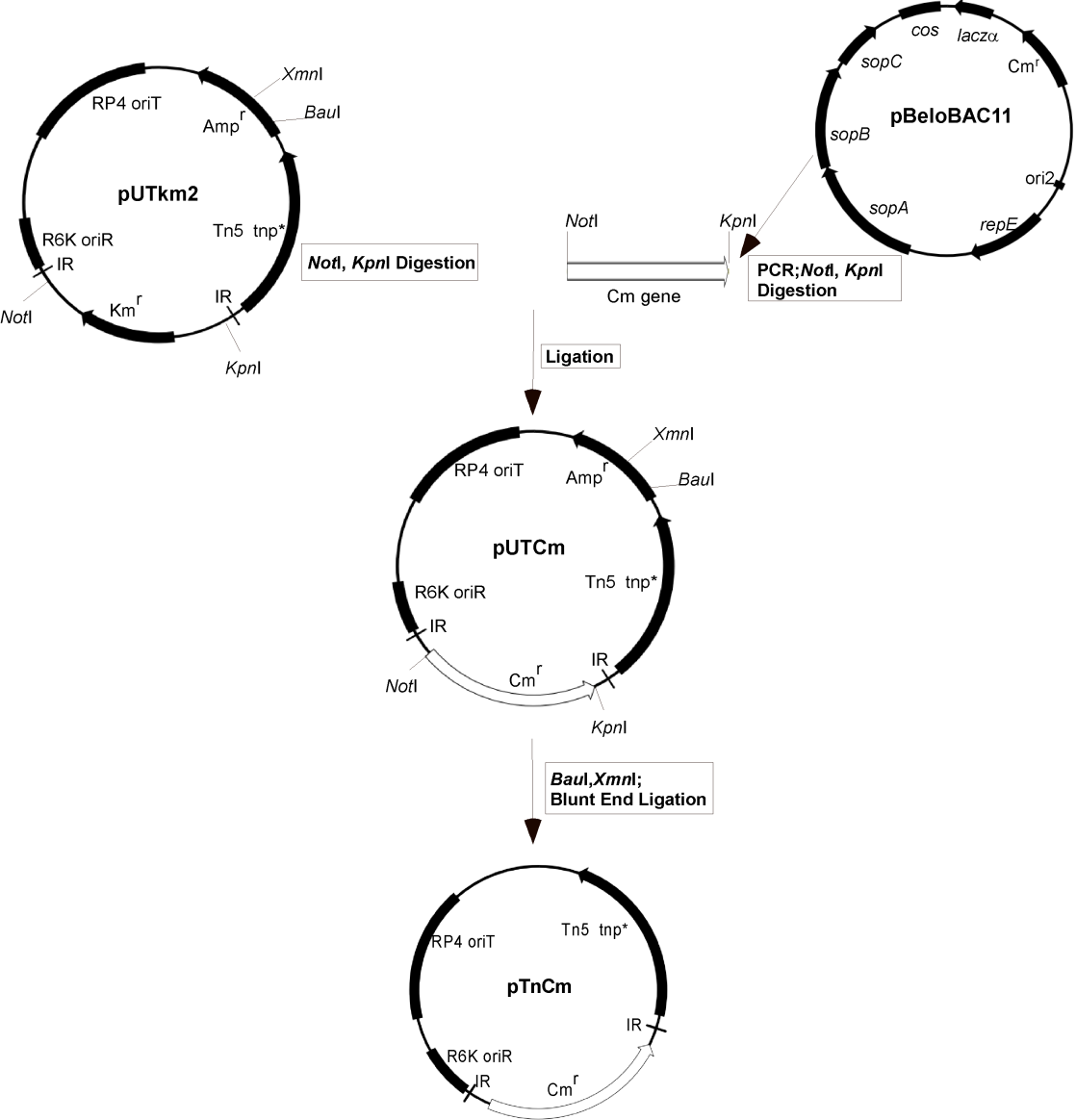


**Figure S1. Construction of pTnCm.** Arrows and boxed text indicate the construction manipulations. The chloramphenicol resistance cassette from pBeloBAC11 is represented by an open arrow. All plasmids are drawn to scale. IR, inverted repeat.
