# Supplementary material for: Identification and Molecular Characterization of the Homogentisate Pathway Responsible for Pyomelanin Production, the Major Melanin Constituents in Aeromonas media WS
Source: PLoS One. 2015 Mar 20;10(3):e0120923. doi: 10.1371/journal.pone.0120923 (PMC4368426; doi:10.1371/journal.pone.0120923)
Supplement: S2 Fig — (A) Photographs of cultures from 72 h LB cultures of wild-type A. media strain WS and the 14 transposon mutants (WS-M1, WS-M2, WS-M3, WS-M4, WS-M5, WS-M6, WS-M7, WS-M8, WS-M9, WS-M10, WS-M11, WS-M12, WS-M13, WS-M14). (B) Wild-type A. media strain WS and the 14 transposon mutants were cultured in LB, and then at 72 h post-inoculation, the OD400 of the cultures were determined. (C) Wild-type A. media strain WS and the 14 transposon mutants were cultured in LB, and then at 72 h post-inoculation, the OD600 of the cultures were determined. (DOC) [file pone.0120923.s002.doc]

**Figure S2. 14 mutants with attenuated melanin production.**

**
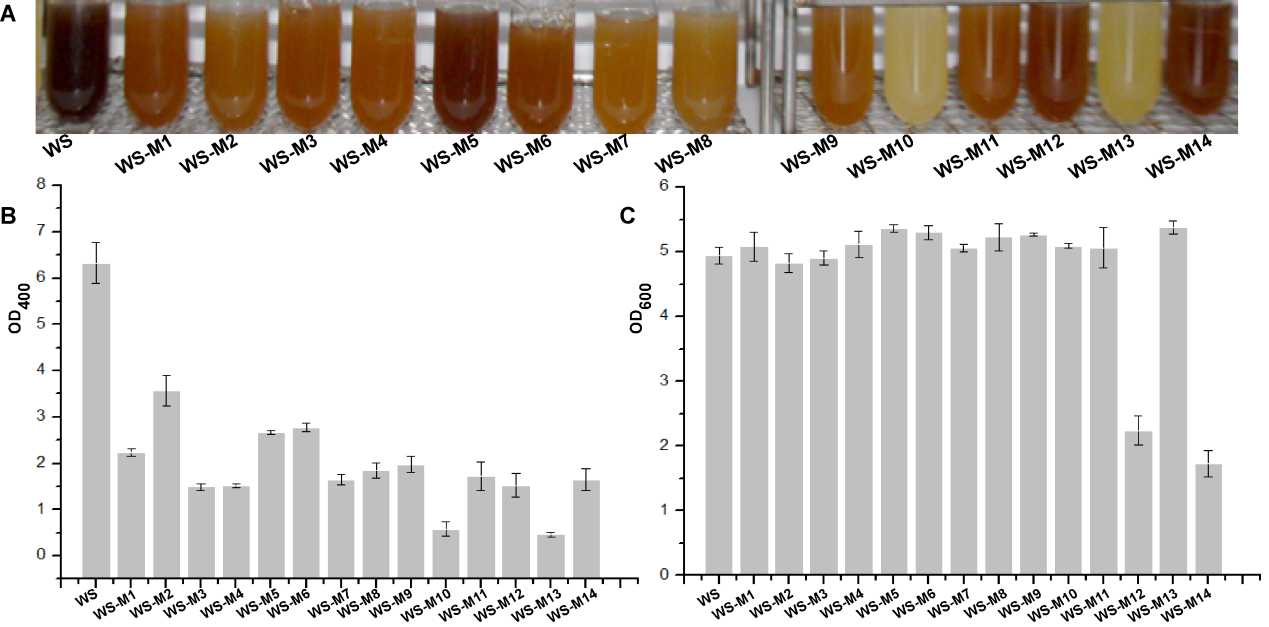
**

**Figure S2.** **14 mutants with attenuated melanin production.** (A) Photographs of cultures from 72 h LB cultures of wild-type *A*. *media* strain WS and the 14 transposon mutants (WS-M1, WS-M2, WS-M3, WS-M4, WS-M5, WS-M6, WS-M7, WS-M8, WS-M9, WS-M10, WS-M11, WS-M12, WS-M13, WS-M14). (B) Wild-type *A*. *media* strain WS and the 14 transposon mutants were cultured in LB, and then at 72 h post-inoculation, the OD_400_ of the cultures were determined. (C) Wild-type *A*. *media* strain WS and the 14 transposon mutants were cultured in LB, and then at 72 h post-inoculation, the OD_600_ of the cultures were determined.
