# Supplementary material for: Identification and Molecular Characterization of the Homogentisate Pathway Responsible for Pyomelanin Production, the Major Melanin Constituents in Aeromonas media WS
Source: PLoS One. 2015 Mar 20;10(3):e0120923. doi: 10.1371/journal.pone.0120923 (PMC4368426; doi:10.1371/journal.pone.0120923)
Supplement: S3 Fig — Detection of HGA from cultures of wild-type A. media strain WS and non-pigmented mutants WS-M10 and WS-M13. Samples were taken at 12-h intervals until 72 h at 30°C in LB. (A) Analysis of the culture from wild-type A. media strain WS. (B) Analysis of the culture from non-pigmented mutant WS-M10. (C) Analysis of the culture from non-pigmented mutant WS-M13. The asterisk indicates the peak of HGA. (DOC) [file pone.0120923.s003.doc]

**Figure S3. HPLC analysis.**

**
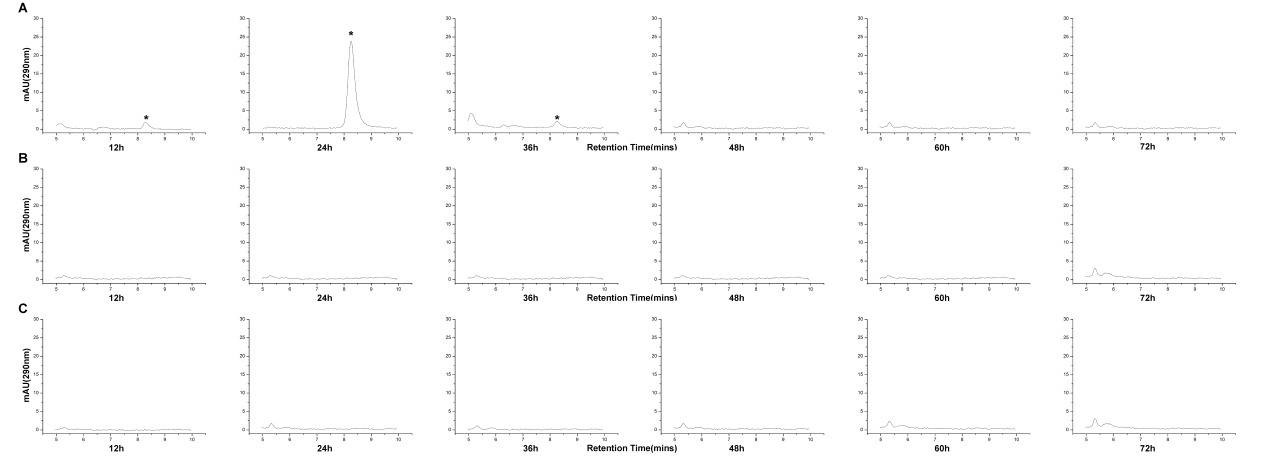
**

**Figure S3. HPLC analysis.** Detection of HGA from cultures of wild-type *A. media* strain WS and non-pigmented mutants WS-M10 and WS-M13. Samples were taken at 12-h intervals until 72 h at 30℃ in LB. (A) Analysis of the culture from wild-type *A. media* strain WS. (B) Analysis of the culture from non-pigmented mutant WS-M10. (C) Analysis of the culture from non-pigmented mutant WS-M13. The asterisk indicates the peak of HGA.
