# Supplementary material for: Identification and Molecular Characterization of the Homogentisate Pathway Responsible for Pyomelanin Production, the Major Melanin Constituents in Aeromonas media WS
Source: PLoS One. 2015 Mar 20;10(3):e0120923. doi: 10.1371/journal.pone.0120923 (PMC4368426; doi:10.1371/journal.pone.0120923)
Supplement: S4 Fig — (A) MS analysis of an authentic L-DOPA sample. (B) MS analysis of the sample from culture of wild-type A. media strain WS. (C) MS analysis of an authentic HGA sample. (D) MS analysis of the sample from culture of wild-type A. media strain WS. (DOC) [file pone.0120923.s004.doc]

**Figure S4. MS analysis.**

**
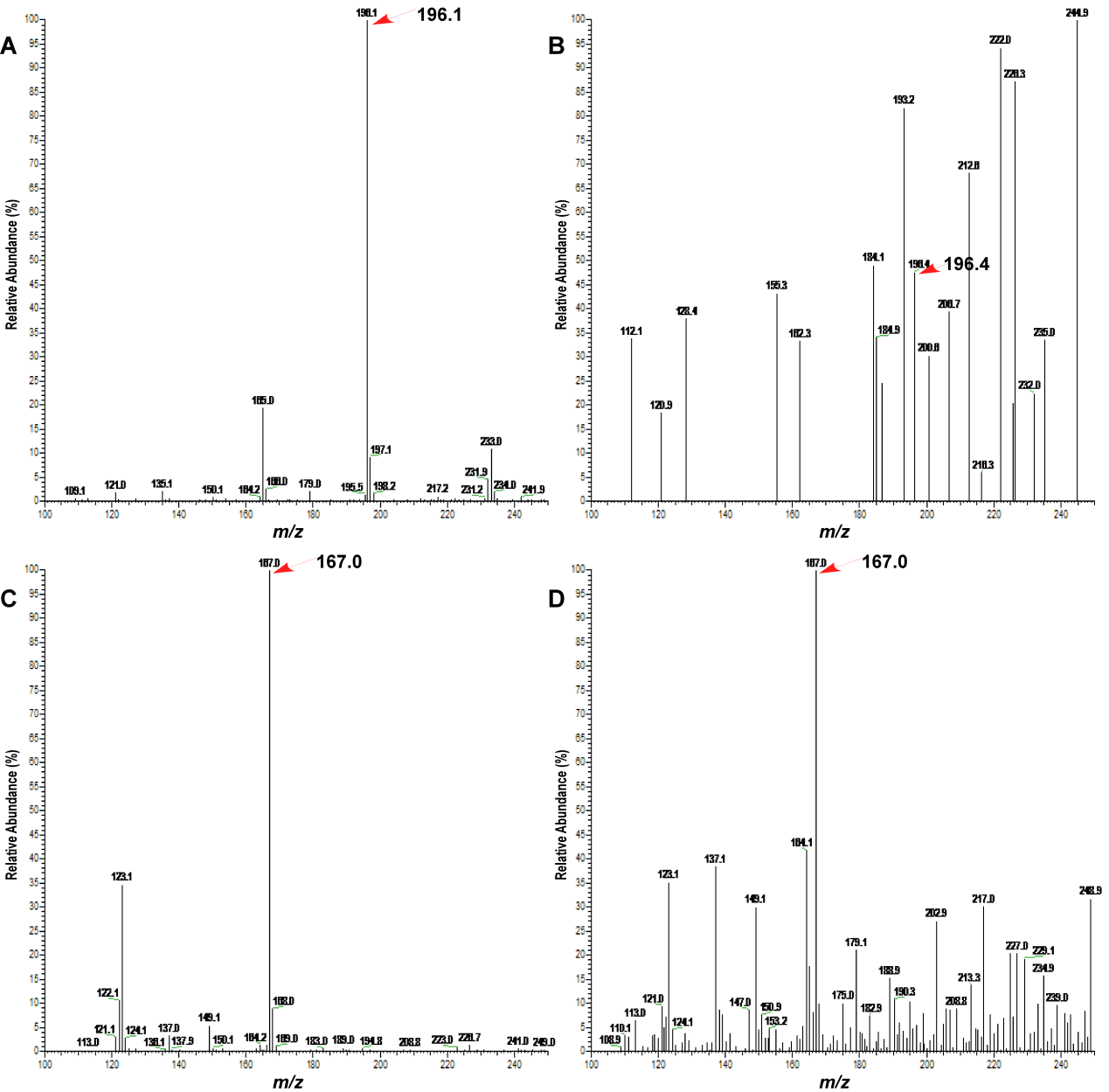
**

**Figure S4. MS analysis.** (A) MS analysis of an authentic L-DOPA sample. (B) MS analysis of the sample from culture of wild-type *A. media* strain WS. (C) MS analysis of an authentic HGA sample. (D) MS analysis of the sample from culture of wild-type *A. media* strain WS.
