# Supplementary material for: Identification and Molecular Characterization of the Homogentisate Pathway Responsible for Pyomelanin Production, the Major Melanin Constituents in Aeromonas media WS
Source: PLoS One. 2015 Mar 20;10(3):e0120923. doi: 10.1371/journal.pone.0120923 (PMC4368426; doi:10.1371/journal.pone.0120923)
Supplement: S5 Fig — The primary structure and conservation of HppD from A. hydrophila_XS91-4-1 compared to that from A. hydrophila ML09-119, A. hydrophila ATCC7966, A. hydrophila 4AK4. Amino acids depicted in gray are little conserved. (DOC) [file pone.0120923.s005.doc]

**Figure S5. HppD primary structure.**

**
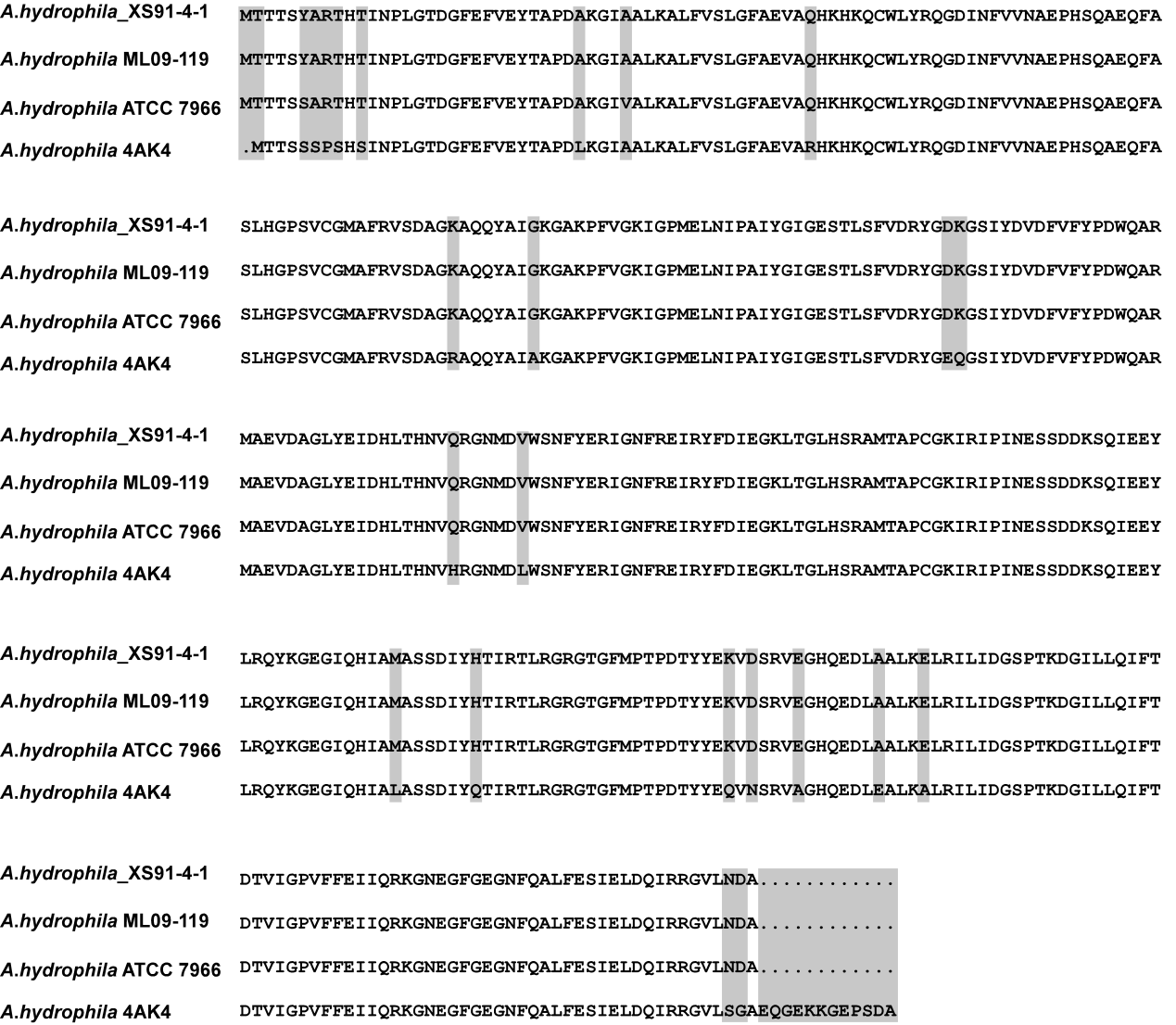
**

**Figure S5.** **HppD primary structure.** The primary structure and conservation of HppD from *A. hydrophila*_XS91-4-1 compared to that from *A. hydrophila* ML09-119, *A. hydrophila* ATCC7966, *A. hydrophila* 4AK4. Amino acids depicted in gray are little conserved.
