# Supplementary material for: Identification and Molecular Characterization of the Homogentisate Pathway Responsible for Pyomelanin Production, the Major Melanin Constituents in Aeromonas media WS
Source: PLoS One. 2015 Mar 20;10(3):e0120923. doi: 10.1371/journal.pone.0120923 (PMC4368426; doi:10.1371/journal.pone.0120923)
Supplement: S6 Fig — Expression of phhA, phhB, tyrB, aspC and hppD transcripts in A. salmonicida_AB98041, A. salmonicida KACC14791, A. hydrophila_XS94-1-4 and A. media strain WS, which were cultivated in LB at 30°C for 12, 24, 48 and 72 h cultivation. And then RT-PCR was done using primers that amplify the specific transcripts. That the PCR products obtained resulted from mRNA templates was confirmed by the lack of product obtained when the PCR did not incorporate reverse transcriptase (- RT). PCR products obtained from genomic DNA appear in the left-most lane, indicating that the mRNAs observed are full-length. RT-PCR analysis of 16S rRNA served as a positive control. (DOC) [file pone.0120923.s006.doc]

**Figure S6. Transcription of *phhA*, *phhB*, *tyrB*, *aspC* and *hppD*.**

**
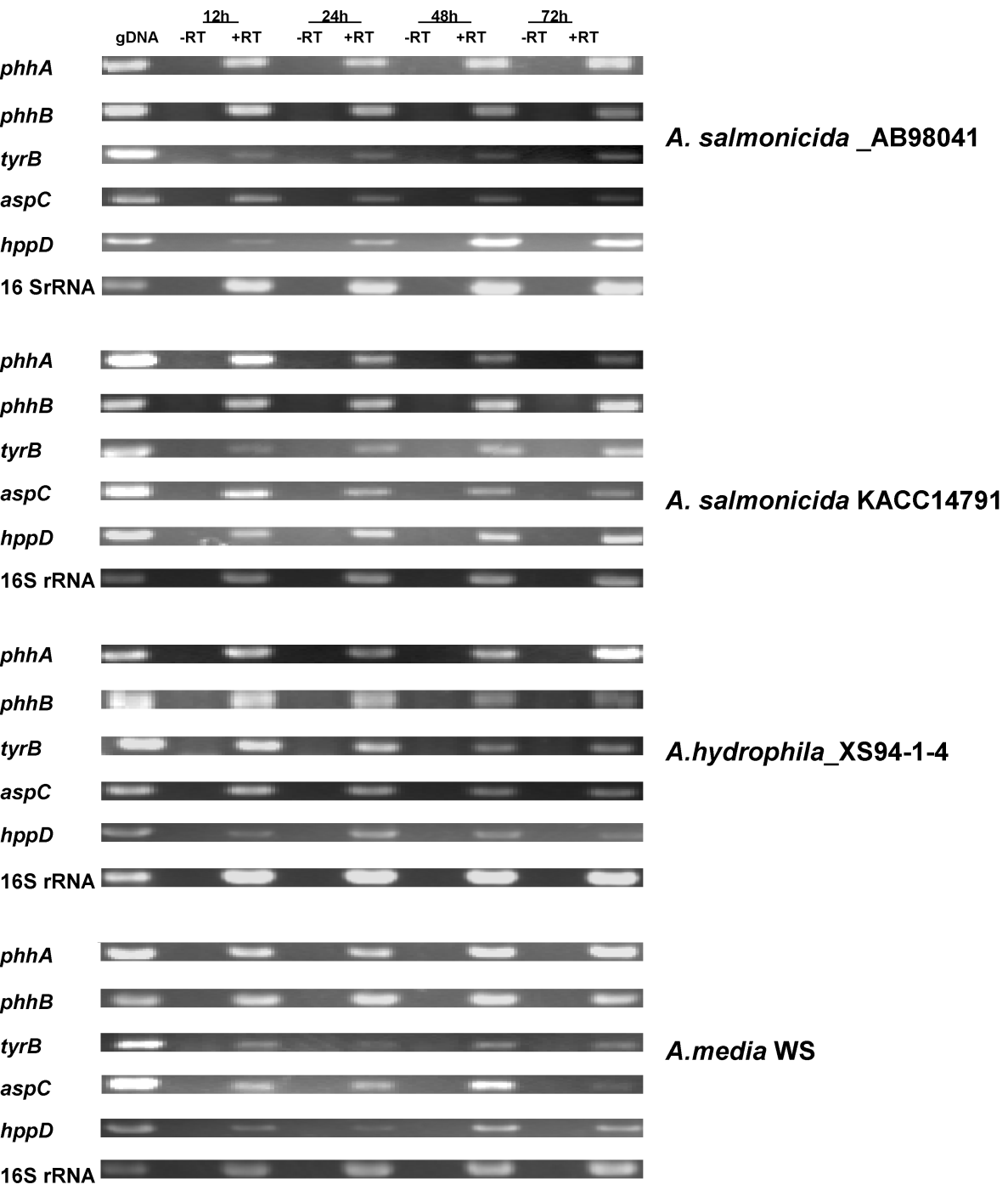
**

**Figure S6. Transcription of *phhA*, *phhB*, *tyrB*, *aspC* and *hppD*.** Expression of *phhA*, *phhB*, *tyrB*, *aspC* and *hppD* transcripts in *A. salmonicida*_AB98041, *A. salmonicida* KACC14791, *A. hydrophila*_XS94-1-4 and *A. media* strain WS, which were cultivated in LB at 30℃ for 12, 24, 48 and 72 h cultivation. And then RT-PCR was done using primers that amplify the specific transcripts. That the PCR products obtained resulted from mRNA templates was confirmed by the lack of product obtained when the PCR did not incorporate reverse transcriptase (- RT). PCR products obtained from genomic DNA appear in the left-most lane, indicating that the mRNAs observed are full-length. RT-PCR analysis of 16S rRNA served as a positive control.
