# Supplementary material for: Identification and Molecular Characterization of the Homogentisate Pathway Responsible for Pyomelanin Production, the Major Melanin Constituents in Aeromonas media WS
Source: PLoS One. 2015 Mar 20;10(3):e0120923. doi: 10.1371/journal.pone.0120923 (PMC4368426; doi:10.1371/journal.pone.0120923)
Supplement: S7 Fig — (A) The hmgA gene in A. media strain WS is interrupted into two parts, hmgA1 and hmgA2. hmgA, gene encoding the homogentisate dioxygenase. B224_2888, gene encoding a transposase. (B) Wild-type A. media strain WS, WSΔhmgA1, WSΔhmgA2 were cultured in LB, and then at 72 h and 96 h post-inoculation, the OD400 of the cultures were determined. (C) Wild-type A. media strain WS, WS (pBBR1MCS-5), WS (pBBR1MCS-5-hmgA-AS), WS (pBBR1MCS-5-hmgA-KACC) and WS (pBBR1MCS-5-hmgA-AH) were cultured in LB, and then at 72 h and 96 h post-inoculation, the OD400 of the cultures were determined. (DOC) [file pone.0120923.s007.doc]

**Figure S7. The function of *hmgA* in pigmentation in *A. media* WS.**


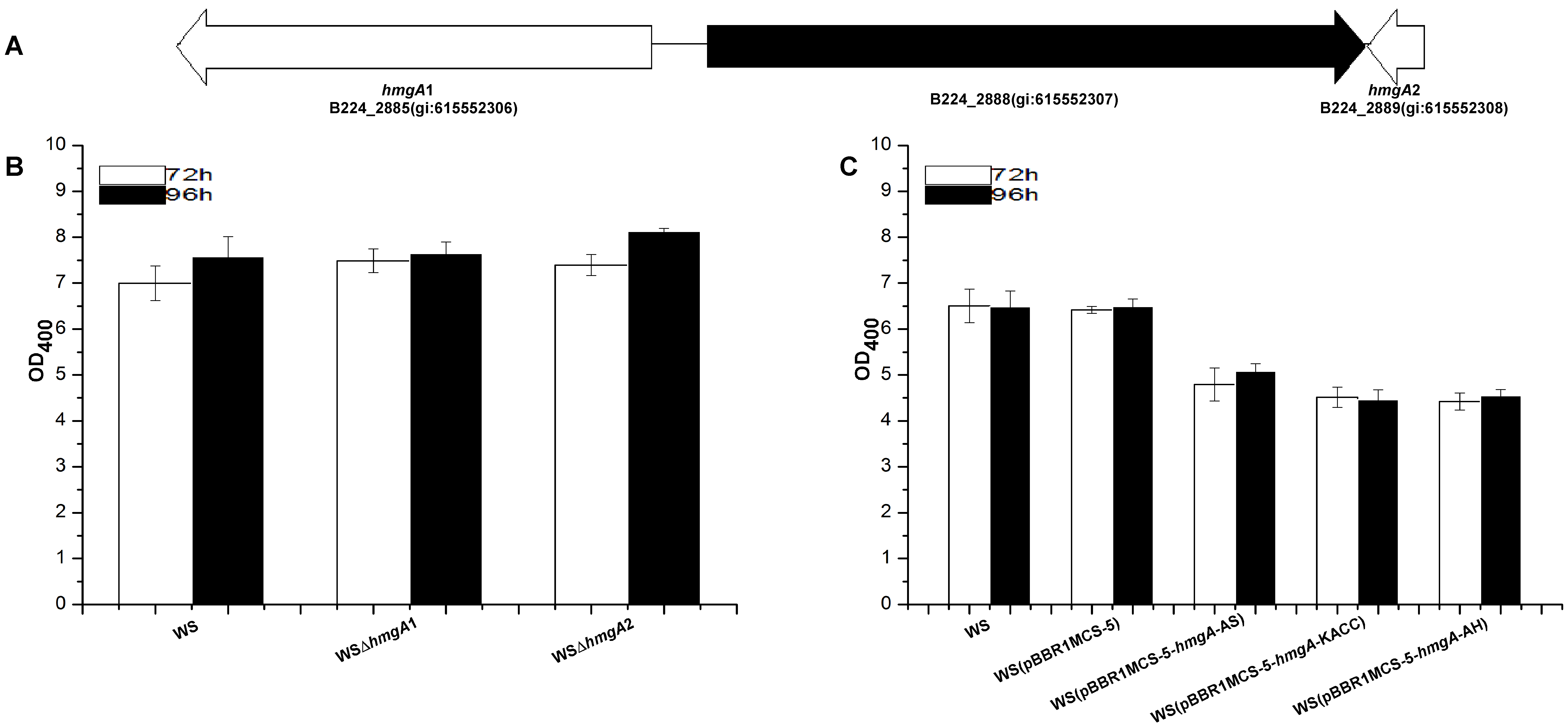


**Figure S7. The function of *hmgA* in pigmentation in *A. media* WS.** (A) The *hmgA* gene in *A. media* strain WS is interrupted into two parts, *hmgA1* and *hmgA2*. *hmgA*, gene encoding the homogentisate dioxygenase. B224_2888, gene encoding a transposase. (B) Wild-type *A. media* strain WS, WS∆*hmgA1*, WS∆*hmgA2* were cultured in LB, and then at 72 h and 96 h post-inoculation, the OD_400_ of the cultures were determined. (C) Wild-type *A. media* strain WS, WS (pBBR1MCS-5), WS (pBBR1MCS-5-*hmgA-*AS), WS (pBBR1MCS-5-*hmgA-*KACC) and WS (pBBR1MCS-5-*hmgA-*AH) were cultured in LB, and then at 72 h and 96 h post-inoculation, the OD_400_ of the cultures were determined.
