# Supplementary material for: Identification and Molecular Characterization of the Homogentisate Pathway Responsible for Pyomelanin Production, the Major Melanin Constituents in Aeromonas media WS
Source: PLoS One. 2015 Mar 20;10(3):e0120923. doi: 10.1371/journal.pone.0120923 (PMC4368426; doi:10.1371/journal.pone.0120923)
Supplement: S1 Table — (DOC) [file pone.0120923.s008.doc]

**Table S1.** Primers used in this study.

| Primer | Sequence^a^(5'→3') | Use |
| --- | --- | --- |
| Used in TAIL-PCR |  |  |
| SP1 | TTACAACAGTACTGCGATGAGTGGC | Identification of Tn5 transposon insertion sites (specific primer) |
| SP2 | TGGTTGCTACGCCTGAATAAGTGAT | Identification of Tn5 transposon insertion sites (specific primer) |
| SP3 | GGCAGAAATTCGATGATAAGCTGTC | Identification of Tn5 transposon insertion sites (specific primer) |
| AD1 | NTCgA(g/C)T(A/T)T(g/C)g(A/T)gTT | Identification of Tn5 transposon insertion sites (arbitrary degenerate primer) |
| AD2 | NgTCgA(g/C)(A/T)gANA(A/T)Gaa | Identification of Tn5 transposon insertion sites (arbitrary degenerate primer) |
| AD3 | (A/T)gTgNAg(A/T)ANCANAgA | Identification of Tn5 transposon insertion sites (arbitrary degenerate primer) |
| AD4 | Tg(A/T)gNAg(A/T)ANCA(g/C)AgA | Identification of Tn5 transposon insertion sites (arbitrary degenerate primer) |
| AD5 | Ag(A/T)gNAg(A/T)ANCA(A/T)Agg | Identification of Tn5 transposon insertion sites (arbitrary degenerate primer) |
| AD6 | CA(A/T)CgICNgAIA(G/C)Gaa | Identification of Tn5 transposon insertion sites (arbitrary degenerate primers) |
| AD7 | TC(g/C)TICgNACIT(A/T)ggA | Identification of Tn5 transposon insertion sites (arbitrary degenerate primer) |
|  |  |  |
| Used in mutagenesis of *phhA* of *A. media* strain WS |  |  |
| phhA(S)-S | ATTGTCGACTCAGCTCGCCTGCCGGCC | Deletion of *phhA* gene |
| phhA(S)-A | GCCTCTAGACCATCGGCGAGACCGCCT | Deletion of *phhA* gene |
| phhA(X)-S | ATTTCTAGAAGGCACGGCGGCCACCG | Deletion of *phhA* gene |
| phhA(X)-A | GCCGAGCTCATGGCAGGGATCATGGC | Deletion of *phhA* gene |
|  |  |  |
| Used in mutagenesis of *tyrB*  of *A. media* strain WS |  |  |
| tyrB(S)-S | GCCGTCGACGCCTATGCTGGCGACCCCA | Deletion of *tyrB* gene |
| tyrB(S)-A | ATTTCTAGAGGTGCGGTAGTTGGCGGCCCC | Deletion of *tyrB* gene |
| tyrB(X)-S | ATTTCTAGACGCATCAAGGCCATGCGC | Deletion of *tyrB* gene |
| tyrB(X)-A | GCCGCATGCTCGACGTTGCGGCTGTTGA | Deletion of *tyrB* gene |
|  |  |  |
| Used in mutagenesis of *aspC* of *A. media* strain WS |  |  |
| aspC(S)-S | ATTGTCGACTGCCGCCCCAGCGGATCCG | Deletion of *aspC* gene |
| aspC(S)-A | GCCTCTAGACGGCCATATTCGATATTGCC | Deletion of *aspC* gene |
| aspC(X)-S | ATTTCTAGAGCGCATTCGCGAGATGCGC | Deletion of *aspC* gene |
| aspC(X)-A | GCCGCATGCGCACAGGGGATCTATGTTGG | Deletion of *aspC* gene |
|  |  |  |
| Used in mutagenesis of *hppD* of *A. media* strain WS |  |  |
| hppD(S)-S | GCCGTCGACGGCACCGATGGGTTCGAA | Deletion of *hppD* gene |
| hppD(S)-A | ATTTCTAGATGCTGGGCACGGCCCGCA | Deletion of *hppD* gene |
| hppD(X)-S | GCCTCTAGACTGGAGGCACTCAAGGCG | Deletion of *hppD* gene |
| hppD(X)-S | ATTGAGCTCGCTCGGCTCCCCCTTCTT | Deletion of *hppD* gene |
|  |  |  |
| Used in mutagenesis of *hmgA1* of *A. media* strain WS |  |  |
| hmgA1(S)-S | ATTGTCGACCTGGTCGCTGCCAGCTGGA | Deletion of *hmgA1* gene |
| hmgA1(S)-A | GCCTCTAGATTCTTCAGCCGGGACAACAT | Deletion of *hmgA1* gene |
| hmgA1(X)-S | ATTTCTAGAAAACTCCCCGCCCCCCTTG | Deletion of *hmgA1* gene |
| hmgA1(X)-A | GCCGCATGCCGACTGGAGCAGTTGGGAGG | Deletion of *hmgA1* gene |
|  |  |  |
| Used in mutagenesis of *hmgA2* of *A. media* strain WS |  |  |
| hmgA2(S)-S | GCCGTCGACCTGCAGATCTTCACCGACA | Deletion of *hmgA2* gene |
| hmgA2(X)-A | ATTTCTAGACCCGGTGGGGGAAACTTAT | Deletion of *hmgA2* gene |
| hmgA2(S)-S | ATTTCTAGAGCCCGAGCAGGCCATCTA | Deletion of *hmgA2* gene |
| hmgA2(X)-A | GCCGAGCTCGTTCCCGGAGGAGTTGGTG | Deletion of *hmgA2* gene |
|  |  |  |
| Used in construction of complementary plasmids |  |  |
| PhhA-S | ATTGTCGACGGCAAGCCGTTACAGTGATCC | Complementation of strain  WS∆*phhA* |
| PhhA-A | GCCGGATCCGTTCAGACATTCGGGACTCC | Complementation of strain  WS∆*phhA* |
| Phh(A+B)-S | ATTGGATCCGCAGGCCCTGTTTCTTCGCGTC | Complementation of strain  WS∆*phhA* |
| Phh(A+B)-A | GCCGTCGACGGCAAGCCGTTACAGTGATC | Complementation of strain  WS∆*phhA* |
| TyrB-S | ATTAAGCTTATAACGGCCCCGGCCCCGGGCC | Complementation of strain  WS∆*tyrB/* WS∆*tyrB*∆*aspC* |
| TyrB-A | GCCGAATTCTCAGCCCTTGAGCACGGCGGCC | Complementation of strain  WS∆*tyrB/* WS∆*tyrB*∆*aspC* |
| AspC-S | GCCGAATTCGAAGGCAGTGGTATAAAGAGTTG | Complementation of strain WS∆*tyrB*∆*aspC* |
| AspC-A | ATTCTGCAGTTACAGCACCTTGGCGATGGCGT | Complementation of strain WS∆*tyrB*∆*aspC* |
| HppD-S | ATTAAGCTTCCGGTGGCGACTGCCTGTC | Complementation of strain  WS∆*hppD* |
| HppD-A | GCCTCTAGATTACGCATCGCTCGGCTCC | Complementation of strain  WS∆*hppD* |
| HmgA(AS)-S | GCCCTCGAGATGCGTAACTGGATAAG | Complementation of strain  WS (the intact *hmgA* cloned from *A. salmonicida* _AB98041) |
| HmgA(AS)-A | ATTTCTAGATTATTTGCCAGTGGCA | Complementation of strain  WS |
| HmgA(KACC)-S | GCCCTCGAGATGCGTAACTGGATAAG | Complementation of strain  WS (the intact *hmgA* cloned from *A. salmonicida* KACC14791) |
| HmgA(KACC)-A | ATTTCTAGATTATTTGCCAGTGGCA | Complementation of strain  WS |
| HmgA(AH)-S | GCCCTCGAGATGCGCAACTGGATTAAC | Complementation of strain  WS (the intact *hmgA* cloned from *A. hydrophila*_XS91-4-1) |
| HmgA(AH)-A | ATTTCTAGATTATTTGCCGTTCGTGGC | Complementation of strain  WS |
|  |  |  |
| Used in cloning *hppD* genes |  |  |
| AS(hppD)-S | AGCCGGCCCGCCCCAGTTCGC | Cloning *hppD* genes from *A. salmonicida*_AB98041/*A. salmonicida* KACC14791 |
| AS(hppD)-A | GGACCCCGAGAAACTATCTGAA | Cloning *hppD* genes from *A. salmonicida* _AB98041/*A. salmonicida* KACC14791 |
| AH(hppD)-S | GGCAGGTCGGCATGAGCC | Cloning *hppD* genes from *A. hydrophila*_XS91-4-1 |
| AH(hppD)-A | CCCCGGTGTTACCCTCTTCGTT | Cloning *hppD* genes from *A. hydrophila*_XS91-4-1 |
| Used in construction of expression plasmids |  |  |
| hppD(WS)-S | GCCCATATGACGACCTCTTCTTCCCGTTC | Expression the *hppD* cloned from *A. media* strain WS |
| hppD(WS)-A | ATTAAGCTTCGCATCGCTCGGCTCCC | Expression the *hppD* cloned from *A. media* strain WS |
| hppD(AS)-S | GCCCATATGACGACCACTTCCTCCTCTT | Expression the *hppD* cloned from *A. salmonicida* _AB98041/*A. salmonicida* KACC14791 |
| hppD(AS)-A | ATTAAGCTTCGCATCGCTGGGTTCTCCA | Expression the *hppD* cloned from *A. salmonicida* _AB98041/*A. salmonicida* KACC14791 |
| hppD(AH)-S | GCCCATATGACGACCACTTCTTATGCCC | Expression the *hppD* cloned from *A. hydrophila*_XS91-4-1 |
| hppD(AH)-A | ATTAAGCTTCGCATCATTGAGCACCCCA | Expression the *hppD* cloned from *A. hydrophila*_XS91-4-1 |
| Used in analysis of *Aeromonas* strains gene expression |  |  |
| 16SsRNA(S) | GGGTGCAAGCGTTAATCGG | Detection of transcription of 16S rRNA |
| 16sRNA(A) | AGGCACTCCCGCATCTCTG | Detection of transcription of 16S rRNA |
| phhA(RT)-S | GAGGAGCACGGCACCTGGCAGA | Detection of transcription of *phhA* |
| phhA(RT)-A | GTGAACCAGTAGAGCCGCGCCA | Detection of transcription of *phhA* |
| phhB(RT)-S | CAGTGTGAAGCGTGCCGCGCC | Detection of transcription of *phhB* |
| phhB(RT)-A | TCCGTACGTGCCGCCATGAT | Detection of transcription of *phhB* |
| tyrB(RT)-S | TTCGAGGGGGCCGGTATCA | Detection of transcription of *tyrB* |
| tyrB(RT)-A | CTTCATCTGGCCCAGCACC | Detection of transcription of *tyrB* |
| aspC(RT)-S | CCGTCAAATGGTACAAGTAC | Detection of transcription of *aspC* |
| aspC(RT)-A | CACTGACCAGGGTGAAGG | Detection of transcription of *aspC* |

^a^ Restriction sites are underlined.
